# Telocyte-derived exosomes promote angiogenesis and alleviate acute respiratory distress syndrome via JAK/STAT-miR-221-E2F2 axis

**DOI:** 10.1186/s43556-025-00259-6

**Published:** 2025-04-08

**Authors:** Rongrong Gao, Xu Zhang, Huihui Ju, Yile Zhou, Luoyue Yin, Liuke Yang, Pinwen Wu, Xia Sun, Hao Fang

**Affiliations:** 1 Department of Anesthesiology, Zhongshan Hospital, Fudan University, Shanghai, China; 2 Clinical Center for Biotherapy at Zhongshan Hospital, Fudan University, Shanghai, China; 3 NHC Key Lab of Reproduction Regulation, Shanghai Engineering Research Center of Reproductive Health Drug and Devices, Shanghai Institute for Biomedical and Pharmaceutical Technologies, Shanghai, China; 4 Shanghai-MOST Key Laboratory of Health and Disease Genomics, NHC Key Lab of Reproduction Regulation, Shanghai Institute for Biomedical and Pharmaceutical Technologies, Shanghai, China; 5 College of Plant Protection, Nanjing Agricultural University, Nanjing, 210000 China; 6 Department of Anesthesiology, Minhang Hospital, Fudan University, Shanghai, China; 7 Department of Anesthesiology, Shanghai Geriatric Medical Center, Shanghai, China

**Keywords:** Acute respiratory distress syndrome, Telocyte, Exosomal microRNA, MiR-221

## Abstract

Acute respiratory distress syndrome (ARDS) is characterized by severe respiratory failure and significant inflammation, leading to vascular and epithelial cell damage. The absence of effective pharmacologic treatments underscores the need for novel therapeutic approaches. Telocytes (TCs), a newly identified type of interstitial cells, have shown potential in tissue repair and angiogenesis, particularly through the release of exosomal microRNAs (miRNAs). Exosomes were isolated from LPS (lipopolysaccharide)-stimulated TCs and characterized using western blotting and nanoparticle tracking analysis. The role of exosomal miR-221 in angiogenesis was assessed through tube formation, migration, and proliferation assays in mouse vascular endothelial cells (MVECs). The JAK/STAT pathway’s involvement in miR-221 regulation was determined using western blotting and qRT-PCR. A dual-luciferase assay confirmed E2F2 as a direct target of miR-221. ARDS mouse model was established via LPS instillation, and the therapeutic effects of TCs-derived exosomes were evaluated by histopathological scoring, cytokine analysis, and endothelial barrier integrity assays. Our findings demonstrated that exosomes from LPS-stimulated TCs significantly promoted angiogenesis, proliferation, and migration in MVECs. These effects were mediated by miR-221, which downregulated E2F2 expression, an important regulator of endothelial cell functions. The JAK/STAT pathway played a crucial role in miR-221 production, with pathway inhibition reducing miR-221 levels and attenuating its pro-angiogenic effects. In vivo, TCs-derived exosomes reduced lung inflammation and tissue damage in ARDS mice, effects that were reversed by miR-221 inhibition. These results suggested that TCs-derived exosomes promoted angiogenesis and alleviated ARDS through the JAK/STAT-miR-221-E2F2 axis.

**Supplementary Information:**

The online version contains supplementary material available at 10.1186/s43556-025-00259-6.

## Introduction

The respiratory system plays a critical role in oxygenating blood and maintaining homeostasis. However, various respiratory conditions, including infections, trauma, and systemic diseases, can severely impair lung function, leading to significant global health challenges [1]. Among these conditions, acute respiratory distress syndrome (ARDS) is a critical syndrome distinguished by the rapid exacerbation of respiratory failure. An international multicentre research indicated that the incidence of ARDS constituted approximately 10.4% of ICU admissions worldwide [2]. The pathogenesis of ARDS involves profound inflammatory responses that lead to significant damage to vascular endothelial and respiratory epithelial cells, severely impairing the lungs’ ability to exchange oxygen and carbon dioxide [3, 4]. The absence of effective therapeutic interventions, coupled with high mortality rates, underscores the urgent need for novel treatment strategies for ARDS. Given its severity and the absence of effective pharmacologic treatments [5], exploring new therapeutic strategies for ARDS is of great importance. Recent studies have highlighted the potential of cell-based therapies, particularly mesenchymal stem cells (MSCs), which have demonstrated efficacy in ameliorating ARDS through paracrine mechanisms [6, 7]. Nonetheless, the clinical application of MSCs is limited by challenges such as the need for fresh sources, costly and time-consuming in vitro expansion, restricted passaging capacity, storage difficulties, and cellular senescence.

Telocytes (TCs) are a newly identified type of interstitial cells characterized with podoms, podomers, and long telopodes (Tp) [8]. TCs have been found in numerous organs/tissues, especially under airway epithelial cells and interstitial tissues of lungs, where they connect with multiple cells for cell-to-cell communication through networks formed by telopodes [9] and interact with stem cells in niches located in the heart [10] and lungs [11]. TCs possess post-injury repair and regeneration functions [12–14], contributing to angiogenesis in both the myocardium [15] and the lungs [16]. Transplantation of cardiac TCs has been reported to promote repair of ischemic myocardium [17], while pulmonary TCs have been found to facilitate angiogenesis and structural formation of the air-blood barrier [16]. Additionally, intratracheal administration of activated TCs has been shown to alleviate ventilator-induced lung injury in a mouse model through releasing angiogenic factors [18]. While intratracheal administration of TCs has shown promise in alleviating lung injury, exosomes derived from TCs may offer additional advantages including enhanced safety, stability and efficient delivery of therapeutic molecules, positioning them as a particularly promising strategy for ARDS treatment.

Exosomes, the lipid bilayer membrane vesicles originating from the luminal membrane of multi-vesicular bodies, were demonstrated to facilitate cell-to-cell communication [19, 20]. Typical exosomes often contain diverse functional mRNAs, microRNAs (miRNAs), as well as proteins, playing a vital role in intercellular communication through the transfer of their genetic contents [21, 22]. The JAK/STAT signaling pathway was found to be a critical regulator of miRNA expression [23, 24], and under inflammatory conditions induced by LPS, activation of the JAK/STAT pathway was accompanied by a significant increase in the expression of miRNAs such as miR-221 in telocytes [25]. MiR-221 play key roles in cancer, cardiovascular and inflammatory diseases [26, 27]. It facilitates tumor growth and chemoresistance in hepatocellular carcinoma by targeting cell cycle inhibitors and pro-apoptotic proteins, and modulates vascular smooth muscle cell proliferation and migration in atherosclerosis [26–28]. Moreover, miR-221-5p targets JNK2, thereby exacerbating lung inflammation and injury during sepsis, underscoring its important role in inflammatory and pulmonary pathologies [29].

E2F2, a member of the E2F transcription factor family, exerts considerable influence on cell cycle progression and apoptosis [30, 31]. Within the scope of ARDS, the functional roles of E2F2 are particularly significant, as the pathogenesis of ARDS is deeply associated with cellular proliferation, inflammation, and vascular damage [3]. E2F2 is notably involved in the regulation of endothelial cell functions, which are critical for maintaining vascular integrity and facilitating angiogenesis. A recent research demonstrated that miR-221 directly targeted E2F2, influencing its expression and activity, which was essential for angiogenesis and endothelial function, processes crucial for repairing vascular damage in ARDS and promoting angiogenesis in endothelial cells within the context of pancreatic ductal adenocarcinoma (PDAC), thereby highlighting miR-221’s role in vascular processes across different diseases [32].

In this study, we focused on the activation of the JAK/STAT pathway in telocytes post-LPS stimulation, which significantly elevated miR-221 expression within exosomes. We further scrutinized the impact of miR-221 on E2F2, investigating its potential role in facilitating vascular regeneration and tissue repair in the context of ARDS.

## Results

### TCs-derived exosomes promote angiogenesis in MVECs under LPS-induced inflammation

**Fig. 1:**
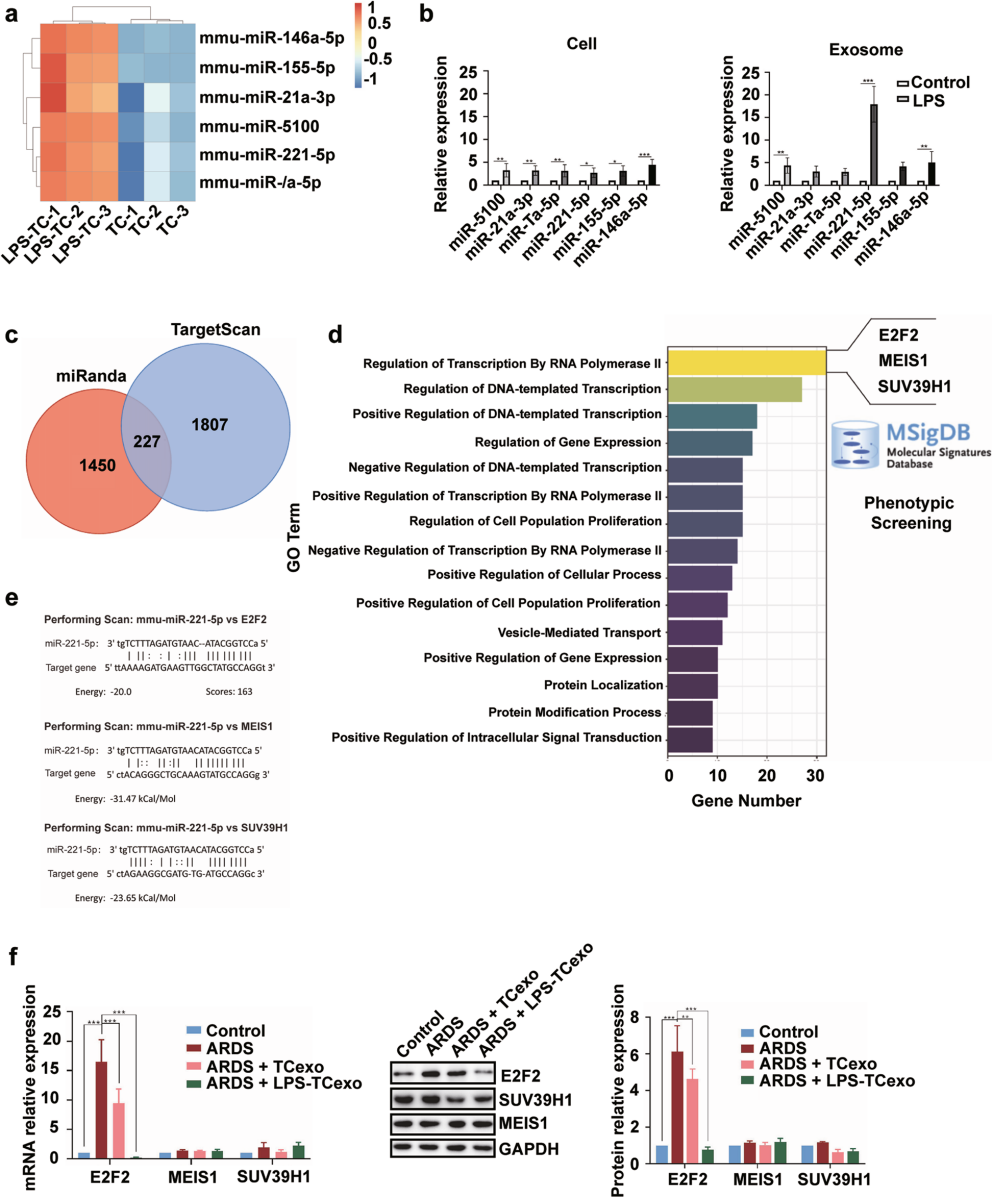
Differential expression and functional analysis of miRNAs and their target genes in TCs and ARDS mouse model. **a** Heat map of differential expressed miRNAs in cultured TCs stimulated with LPS. **b** Expression levels of miRNAs in TCs (top) and TCs-derived exosomes (bottom) measured by qRT-PCR. Control, non-treatment; LPS, LPS stimulation. **c** Venn diagram of the miRNA target genes predicted by miRanda and TargetScan. **d** GO enrichment analysis of 227 overlapping target genes shared by miRanda and TargetScan, of which 3 genes (E2F2, SUV39H1, and MEIS1) were transcription factors involved in vascular repair and cell cycle in the MSigDB database. **e** Target site sequences for miR-221-5p and its target genes E2F2, SUV39H1, and MEIS1. **f** mRNA and protein expression levels of E2F2, MEIS1, and SUV39H1 in lung tissues under different treatments. Control, mice treated with normal saline; ARDS, ARDS mice established by intranasal instillation of LPS; ARDS + TCexo, ARDS mice treated with exosomes derived from TCs; ARDS + LPS-TCexo, ARDS mice treated with exosomes derived from LPS-stimulated TCs. *P < 0.05, **P < 0.01, ***P < 0.001

To identify key miRNAs involved in TC-mediated regulation of ARDS, we first performed miRNA expression profiling in TCs under LPS stimulation. Significant differential expression of miRNAs was revealed in cultured TCs stimulated with LPS, highlighting notable changes in miR-146a-5p, miR-155-5p, miR-21a-3p, miR-5100, miR-221-5p, and miR-7a-5p (Fig. 1a). These differences were further confirmed by quantitative RT-PCR, showing increased expression of miR-5100, miR-221a-5p, and miR-146a-5p in both TCs and TCs-derived exosomes under LPS stimulation, with miR-221-5p exhibiting the most significant upregulation in TCs-derived exosomes (*P* < 0.001, Fig. 1b). A Venn diagram of target genes of miR-221-5p predicted by miRanda and TargetScan identified 227 common targets (Fig. 1c). Gene Ontology (GO) enrichment analysis of these 227 overlapping target genes revealed that 3 genes, E2F transcription factor 2 (E2F2), suppressor of variegation 3–9 homolog 1 (SUV39H1), and Meis homeobox 1 (MEIS1), were transcription factors involved in vascular repair and cell cycle in the MSigDB database, with the predicted binding site sequences of miR-221-5p with E2F2, SUV39H1, and MEIS1 as shown in Fig. 1d and e. In vivo studies using an ARDS mouse model established by intranasal instillation of LPS showed that E2F2 mRNA and protein levels were significantly decreased in ARDS mice treated with exosomes from LPS-stimulated TCs compared to ARDS mice treated with exosomes from TCs and untreated ARDS mice (all *P*-values were significant, Fig. 1f. However, no significant changes were observed in MEIS1 and SUV39H1 expression across the treatment groups (Fig. 1f).

**Fig. 2:**
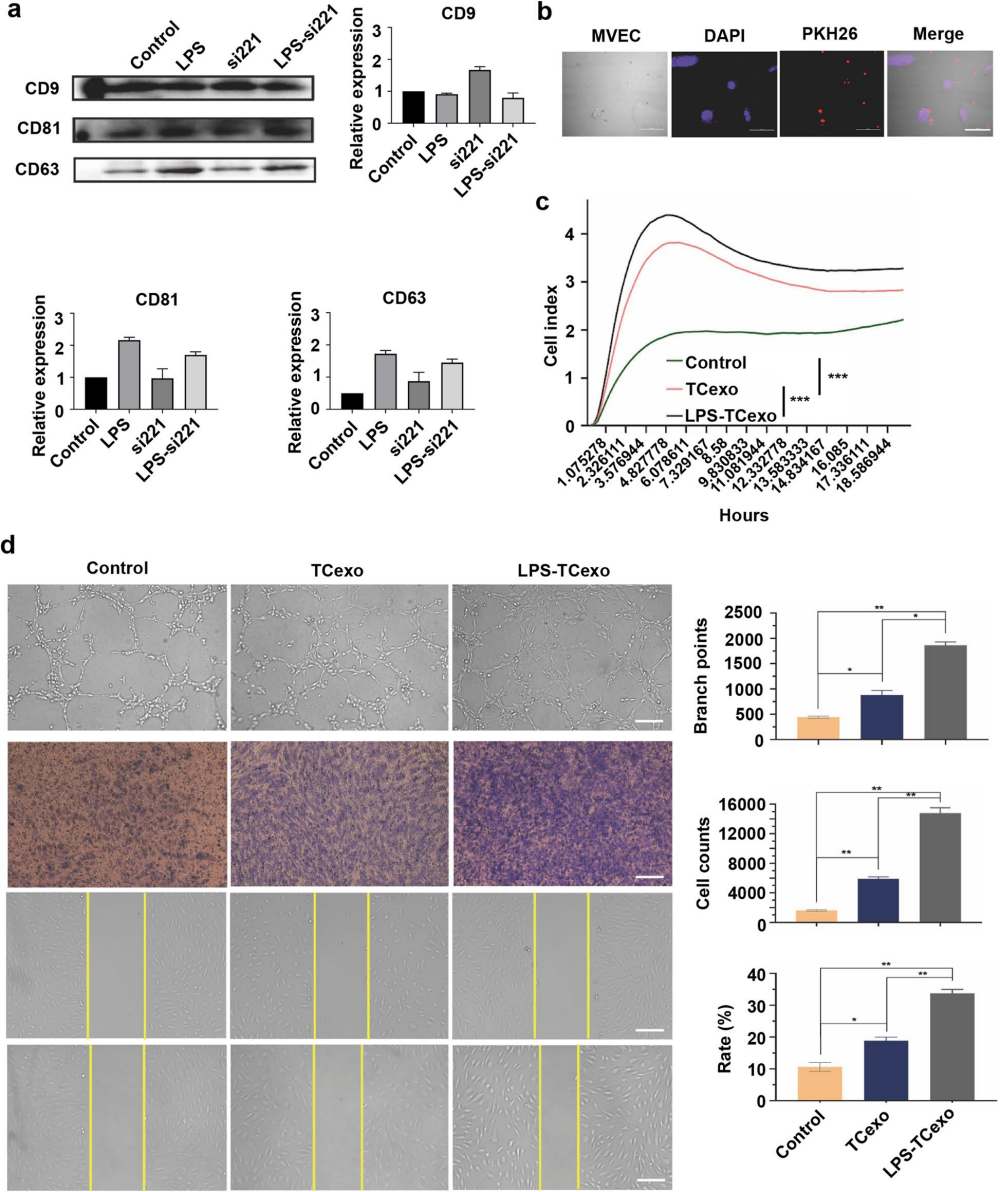
TCs-derived exosomes promote angiogenesis in MVECs under LPS-induced inflammation. **a** Western blot analysis of exosomal markers CD9, CD81, and CD63 in TCs-derived exosomes. Control, untreated TCs; LPS, LPS-stimulated TCs; si221, miR-221 inhibitor-treated TCs; LPS-si221, LPS and miR-221 inhibitor-treated TCs. **b** TCs-derived exosome uptake by MVECs confirmed by immunofluorescence. Blue, DAPI; Red, PKH26. scale bar: 50 μm. **c** The RTCA-based proliferation assay of MVECs. Control, untreated MVECs; TCexo, MVECs incubated with TCs-derived exosomes; TC-LPSexo, MVECs incubated with LfPS-stimulated TCs-derived exosomes. **d** Angiogenesis, proliferation, and migration results of MVECs. Control, untreated MVECs; TCexo, MVECs incubated with TCs-derived exosomes; TC-LPSexo, MVECs incubated with LPS-stimulated TCs-derived exosomes. scale bar: 200 μm. *P < 0.05, **P < 0.01, ***P < 0.001

For exosomes derived from TCs, exosomal markers CD9, CD81, and CD63 were upregulated in response to LPS stimulation, indicating increased exosome secretion. Treatment with a miR-221 inhibitor reduced the expression of these markers, and combined treatment with LPS and the miR-221 inhibitor partially reversed the LPS-induced increase in exosomal marker expression (Fig. 2a). Exosome uptake by MVEC cells was confirmed through PKH26 labeling (Fig. 2b), where red fluorescence indicating exosomes was observed within the cells. The real-time cell analysis confirmed comparable initial cell density and growth patterns across all experimental groups (Fig. S2), providing the basis for the proliferation assay results, which showed that MVEC cells treated with exosomes from TCs (TCexo) exhibited a significantly enhanced proliferation rate compared to the control group, an effect further amplified with exosomes from LPS-stimulated TCs (TC-LPSexo) (all *P*-values were significant, Fig. 2c). Angiogenesis, migration, and proliferation assays further supported these findings. In the angiogenesis assay, the TCexo group, in comparison to the control group, significantly increased tube formation (*P* < 0.05, Fig. 2d) and the TC-LPSexo group exhibited the most extensive tube formation (*P* < 0.01, Fig. 2d). Cell proliferation assays indicated that the TCexo group had a significantly higher cell counts than the control, with the TC-LPSexo group showing the highest counts (all *P*-values were significant, Fig. 2d). Migration assays showed that TCexo and TC-LPSexo treatments significantly accelerated wound closure compared to the control group, with the TC-LPSexo group showing the most pronounced effect (all *P*-values were significant, Fig. 2d).

### TCs-derived exosomal miR-221 promotes angiogenesis in MVECs under LPS-induced inflammation

**Fig. 3:**
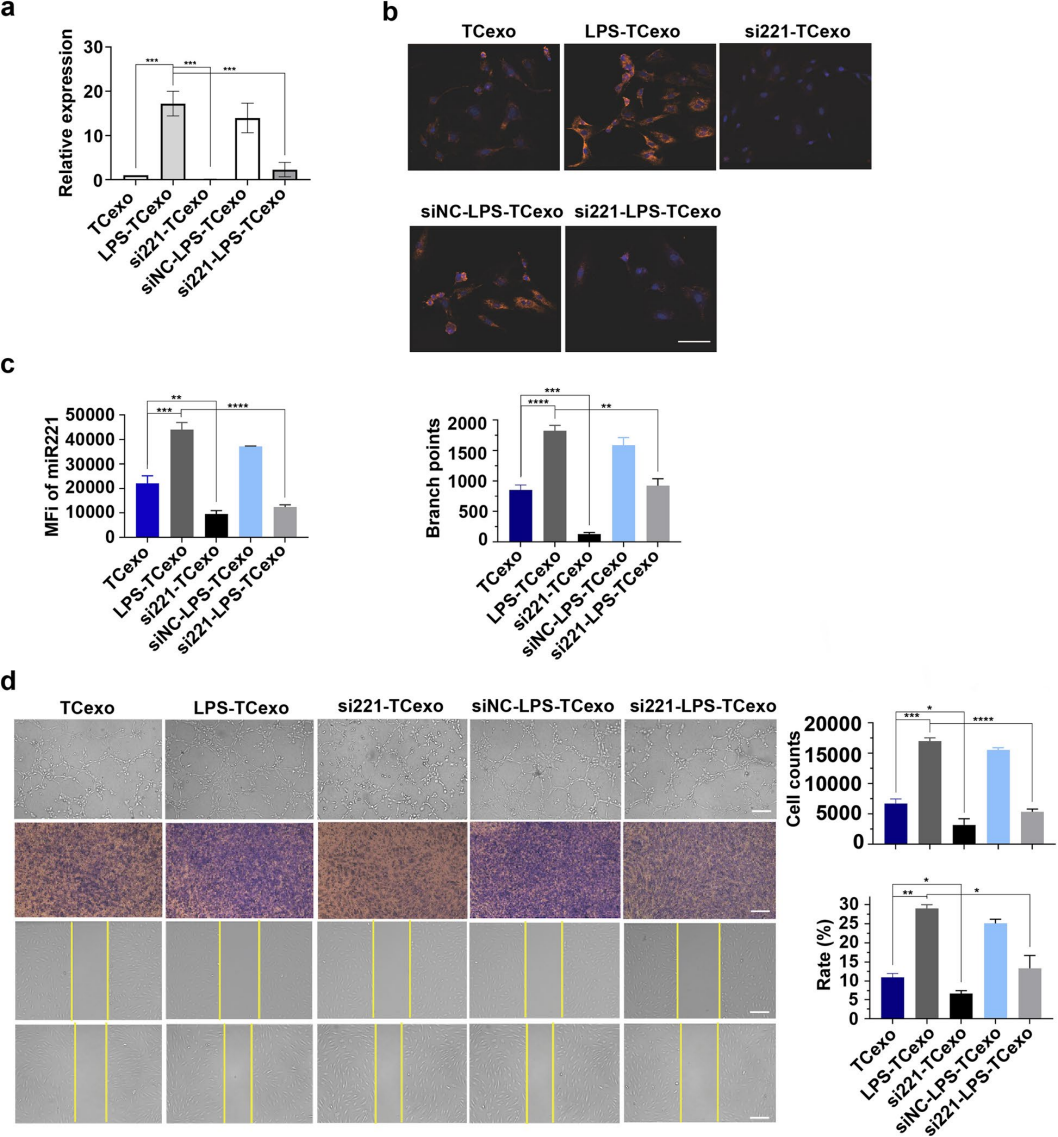
Expression, localization and functional effects of exosomal miR-221 in TCs and MVECs under LPS stimulation. **a** miR-221 expression in TCs-derived exosomes measured by qRT-PCR. TCexo, exosomes derived from untreated TCs; LPS-TCexo, exosomes derived from LPS-stimulated TCs; si221-TCexo, exosomes derived from miR-221 inhibitor-treated TCs; siNC-LPS-TCexo, exosomes derived from LPS and negative control-treated TCs; si221-LPS-TCexo, exosomes derived from LPS and miR-221 inhibitor-treated TCs. **b** Localization of miR-221 within MVECs after incubation with different TCs-derived exosomes. Blue, DAPI; Red, miR-221. TCexo, exosomes derived from TCs; LPS-TCexo, exosomes derived from LPS-stimulated TCs; si221-TCexo, exosomes derived from miR-221 inhibitor-treated TCs; siNC-LPS-TCexo, exosomes derived from LPS and negative control-treated TCs; si221-LPS-TCexo, exosomes derived from LPS and miR-221 inhibitor-treated TCs. scale bar: 50 μm. **c** Fluorescence intensity analysis of miR-221 in MVECs. TCexo, exosomes derived from TCs; LPS-TCexo, exosomes derived from LPS-stimulated TCs; si221-TCexo, exosomes derived from miR-221 inhibitor-treated TCs; siNC-LPS-TCexo, exosomes derived from LPS and negative control-treated TCs; si221-LPS-TCexo, exosomes derived from LPS and miR-221 inhibitor-treated TCs. **d** Angiogenesis, proliferation, and migration results of MVECs incubated with TCs-derived exosomes. Top row: Tube formation assay. Middle row: Crystal violet staining of proliferated cells. Bottom row: Wound healing assay pre- and post-scratch. TCexo, MVECs incubated with TCs-derived exosomes; LPS-TCexo, MVECs incubated with LPS-stimulated TCs-derived exosomes; si221-TCexo, MVECs incubated with miR-221 inhibitor-treated TCs-derived exosomes; siNC-LPS-TCexo, MVECs incubated with LPS and negative control-treated TCs-derived exosomes; si221-LPS-TCexo, MVECs incubated with LPS and miR-221 inhibitor-treated TCs-derived exosomes. scale bar: 200 μm. *P < 0.05, **P < 0.01, ***P < 0.001

To determine the specific contribution of miR-221 to TC-mediated angiogenic effects, we conducted a series of experiments manipulating miR-221 levels in TCs and their derived exosomes. Expression of exosomal miR-221 was significantly upregulated in response to LPS stimulation (*P* < 0.001, Fig. 3a). Treatment with a miR-221 inhibitor effectively reduced miR-221 levels in TCs-derived exosomes, including those from LPS-stimulated TCs (*P* < 0.001, Fig. 3a). miR-221 was found to be localized within MVECs, with increased expression in the LPS-TCexo group, and was significantly reduced by the miR-221 inhibitor even under LPS stimulation, as confirmed by fluorescence intensity analysis (all *P*-values were significant, Fig. 3b, c). Moreover, the significant influence of TCs-derived exosomal miR-221 on the angiogenesis, proliferation, and migration of MVECs was also demonstrated. The tube formation assay showed that exosomes from LPS-stimulated TCs significantly increased the number of branch points compared to the TCexo group, and treatment with miR-221 inhibitor markedly reduced tube formation in si221-TCexo and si221-LPS-TCexo groups (all *P*-values were significant, Fig. 3d). Additionally, cell counts were highest in the LPS-TCexo group, and the application of miR-221 inhibitor resulted in a pronounced decrease in MVEC proliferation (all *P*-values were significant, Fig. 3d). Migration assays indicated that MVECs treated with LPS-TCexo demonstrated the highest wound closure rates, conversely, miR-221 inhibition significantly impaired cell migration (all *P*-values were significant, Fig. 3d).

**Fig. 4:**
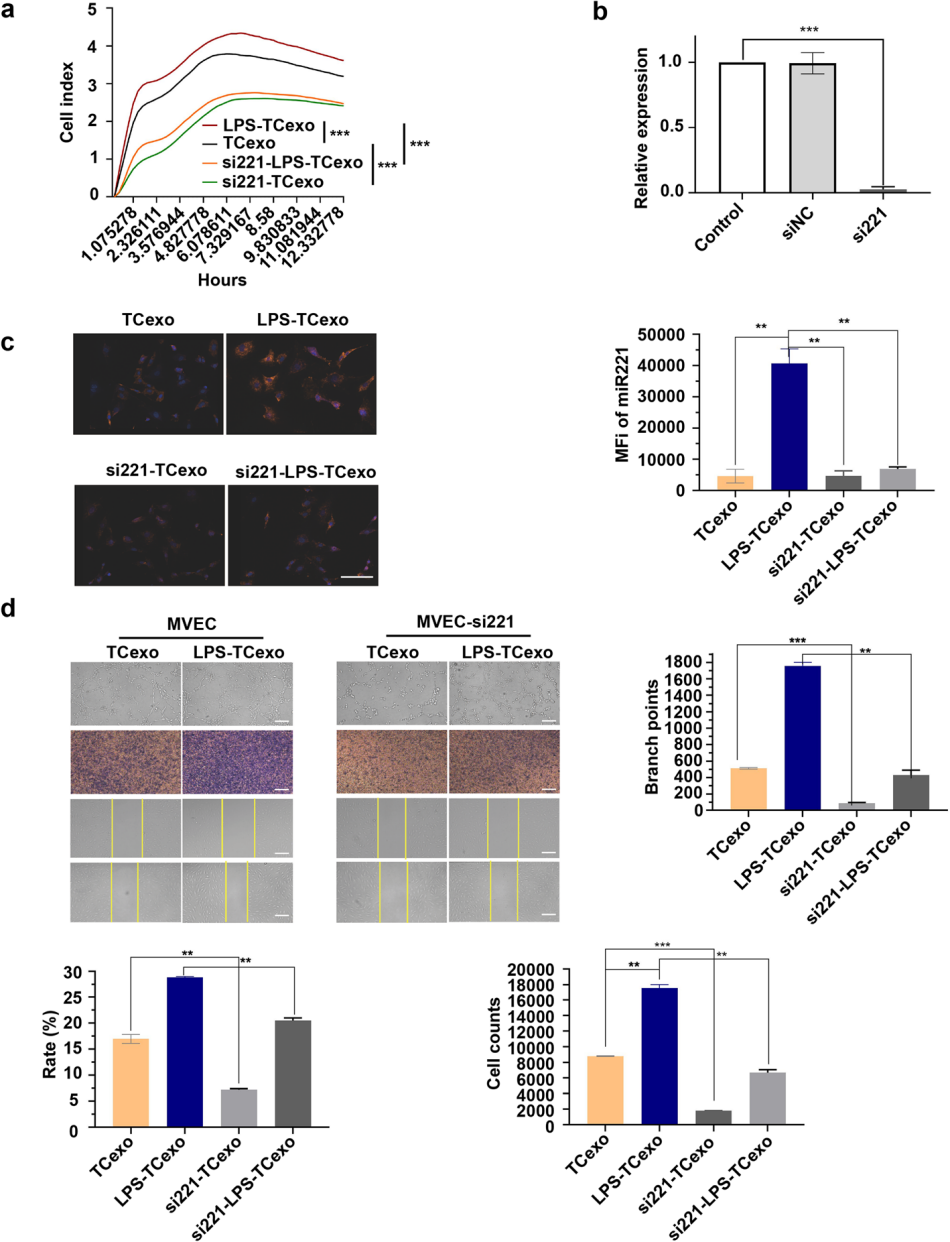
TCs-derived exosomal miR-221 promotes MVEC angiogenesis and proliferation. **a** The RTCA-based proliferation assay of MVECs treated with different TCs-derived exosomes. TCexo, exosomes derived from TCs; LPS-TCexo, exosomes derived from LPS-stimulated TCs; si221/LPS-TCexo, exosomes derived from LPS and miR-221 inhibitor-treated TCs; si221/TCexo: exosomes derived from miR-221 inhibitor-treated TCs. **b** miR-221 expression in MVECs measured by qRT-PCR. Control, untreated MVECs; siNC, negative control-treated MVECs; si221, miR-221 inhibitor-treated MVECs. **c** Localization of miR-221 within MVECs after incubation with different TCs-derived exosomes. Blue, DAPI; Red, miR-221. TCexo, exosomes derived from TCs; LPS-TCexo, exosomes derived from LPS-stimulated TCs; si221-TCexo, exosomes derived from miR-221 inhibitor-treated TCs; si221-LPS-TCexo, exosomes derived from LPS and miR-221 inhibitor-treated TCs. scale bar: 50 μm. **d** Angiogenesis, proliferation, and migration of MVECs and miR-221 knockdown MVECs treated with different TCs-derived exosomes. Top row: Tube formation assay. Middle row: Crystal violet staining of proliferated cells. Bottom row: Wound healing assay pre- and post-scratch. Left: Normal MVECs with control and LPS-TCexo treatments. Right: miR-221 knockdown MVECs with control and LPS-TCexo treatments. Control or TCexo, exosomes derived from TCs; LPS-TCexo, exosomes derived from LPS-stimulated TCs; si221-TCexo, exosomes derived from miR-221 inhibitor-treated TCs; si221-LPS-TCexo, exosomes derived from LPS and miR-221 inhibitor-treated TCs. scale bar: 200 μm. **P < 0.01, ***P < 0.001

To further elucidate the specific roles of miR-221 in MVEC angiogenesis, verify its functional necessity in TCs-derived exosomes, miR-221 inhibition in MVECs was also performed. Results showed that the proliferation was significantly enhanced in MVECs treated with exosomes from LPS-stimulated TCs compared to those treated with exosomes from unstimulated TCs, with this effect markedly reduced upon miR-221 inhibition in both LPS-TCexo and TCexo groups (all *P*-values were significant, Fig. 4a). MiR-221 expression within MVECs was significantly reduced by the miR-221 inhibitor in comparison to the control group (*P* < 0.001, Fig. 4b). Increased miR-221 levels were confirmed in MVECs treated with LPS-TCexo, which were significantly diminished by miR-221 inhibition (*P* < 0.01, Fig. 4c). LPS-TCexo treatment also significantly promoted tube formation, cell proliferation, and migration in MVECs. MVECs following LPS-TCexo treatment exhibited the highest number of branch points and cell counts as well as the fastest wound closure rates, with significant reductions observed in miR-221 knockdown MVECs (all *P*-values were significant, Fig. 4d).

### JAK/STAT pathway mediates TCs-derived exosomal miR-221 in promoting angiogenesis in MVECs

**Fig. 5:**
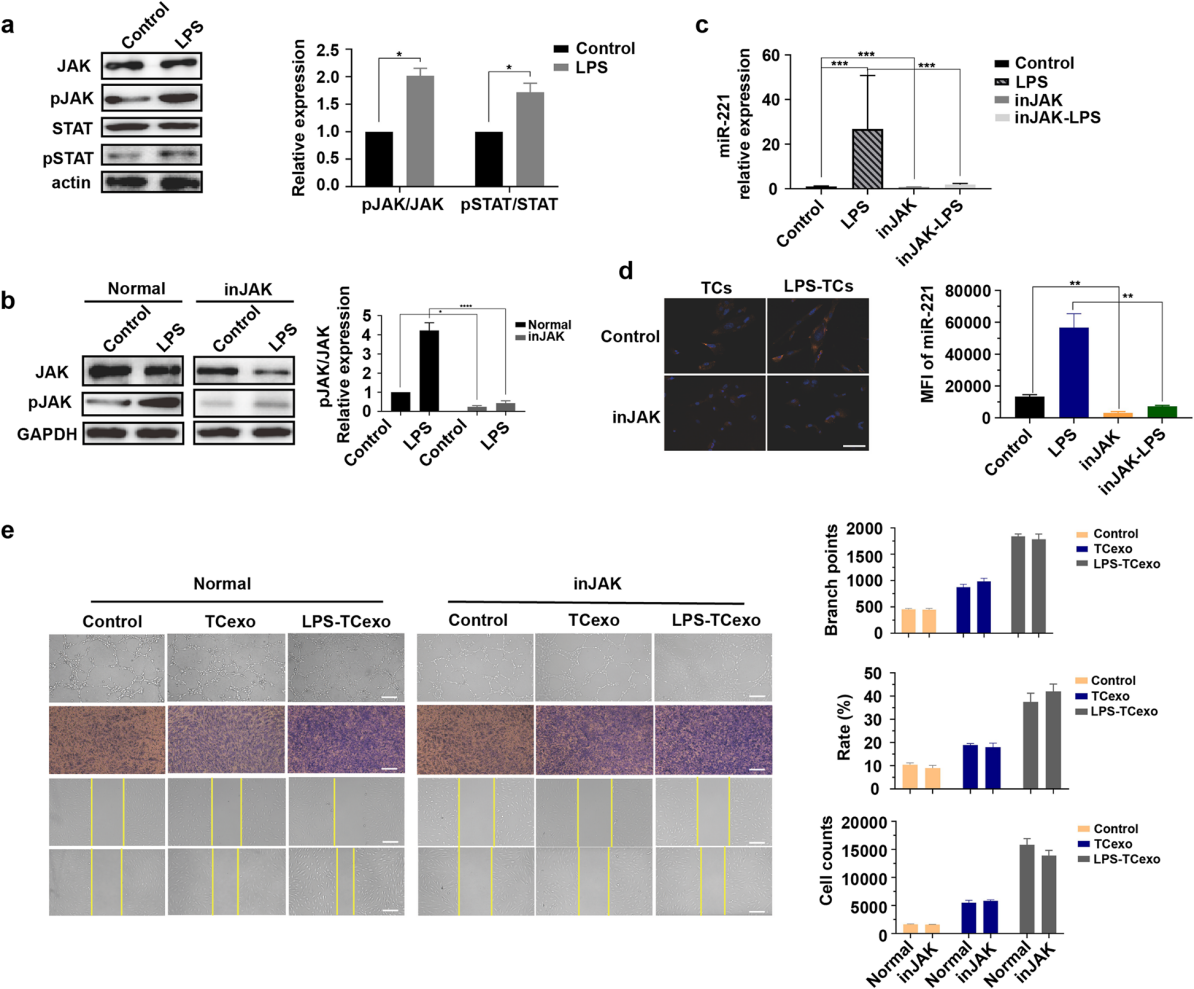
JAK/STAT pathway mediates TCs-derived exosomal miR-221 in promoting angiogenesis in MVECs. **a** Western blot analysis of phosphorylated JAK and STAT in TCs. pJAK, phosphorylated JAK; pSTAT, phosphorylated STAT; Control, untreated TCs; LPS, LPS-stimulated TCs. **b** Western blot analysis of pJAK in TCs with JAK/STAT pathway inhibition. Control, untreated TCs; LPS, LPS-stimulated TCs; inJAK, JAK/STAT pathway- inhibited TCs; inJAK/LPS, JAK/STAT pathway-inhibited and LPS-stimulated TCs. **c** miR-221 expression in TCs measured by qRT-PCR. Control, untreated TCs; LPS, LPS-stimulated TCs; inJAK, JAK/STAT pathway-inhibited TCs; inJAK/LPS, JAK/STAT pathway-inhibited and LPS-stimulated TCs. **d** Immunofluorescence of miR-221 in TCs under different treatments. Blue, DAPI; Red, miR-221. Control, untreated TCs; LPS, LPS-stimulated TCs; inJAK, JAK/STAT pathway-inhibited TCs. scale bar: 50 μm. **e** Angiogenesis, proliferation, and migration of MVECs incubated with different TCs-derived exosomes. Top row: Tube formation assay. Middle row: Crystal violet staining of proliferated cells. Bottom row: Wound healing assay pre- and post-scratch. Control, JAK/STAT pathway uninhibited; TCexo, exosomes derived from TCs; LPS-TCexo, exosomes derived from LPS-stimulated TCs; inJAK-TCexo, exosomes derived from JAK/STAT pathway-inhibited TCs; inJAK/LPS-TCexo, exosomes derived from JAK/STAT pathway-inhibited and LPS-stimulated TCs. scale bar: 200 μm. *P < 0.05, **P < 0.01, ***P < 0.001

To elucidate the upstream regulatory mechanisms controlling miR-221 expression in TCs, we investigated the role of the JAK/STAT pathway. Western blot analysis revealed that LPS stimulation significantly activated the JAK/STAT pathway in TCs, as evidenced by increased phosphorylation levels of JAK and STAT in the LPS-treated group in comparison with the control group (all *P*-values were significant, Fig. 5a). Inhibition of the JAK/STAT pathway effectively reduced the expression of phosphorylated JAK (pJAK), despite the presence of LPS stimulation (all *P*-values were significant, Fig. 5b). MiR-221 expression was significantly elevated in TCs upon LPS stimulation, while JAK/STAT inhibition markedly reduced miR-221 levels, both in the presence and absence of LPS (all *P*-values were significant, Fig. 5c). In situ hybridization revealed that LPS-stimulated TCs exhibited higher miR-221 expression compared to control TCs, whereas inhibition of the JAK/STAT pathway resulted in significantly lower miR-221 levels, even with LPS stimulation (all *P*-values were significant, Fig. 5d). Functionally, exosomes from LPS-stimulated TCs significantly enhanced tube formation, proliferation, and migration of MVECs (all *P*-values were significant, Fig. 5e). These effects were markedly attenuated when the JAK/STAT pathway was inhibited in TCs, demonstrating a reduction in MVEC angiogenesis, proliferation, and migration (all *P*-values were significant, Fig. 5e).

### TCs-derived exosomal miR-221 promotes angiogenesis in MVECs in an E2F2-dependent manner

**Fig. 6:**
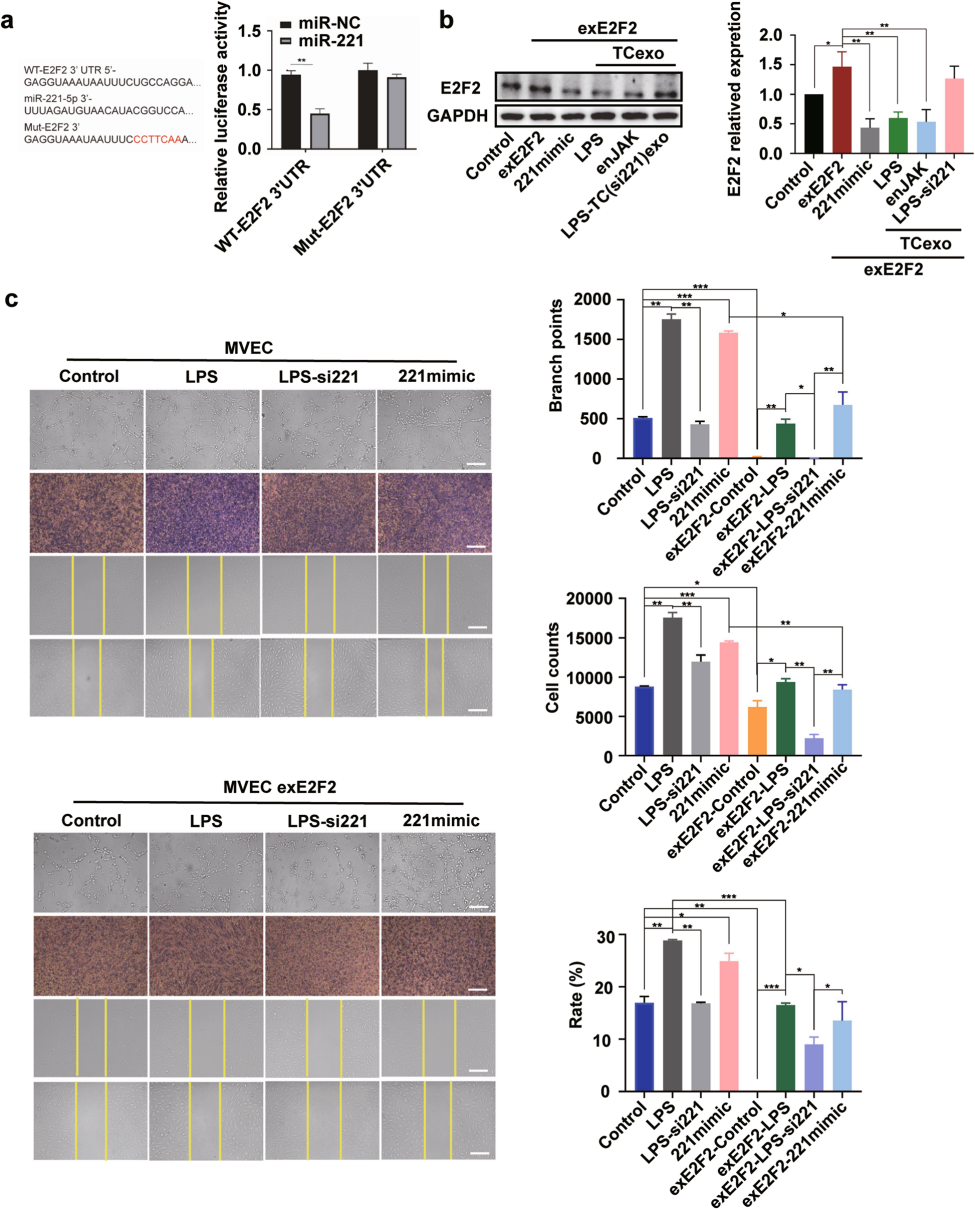
TCs-derived exosomal miR-221 promotes angiogenesis in MVECs in an E2F2-dependent manner. **a** Dual-luciferase reporter assay to test miR-221 binding to wild-type and mutated E2F2 3’ UTR. WT, wild-type; Mut, mutated; miR-NC, negative control. **b** Western blot analysis of E2F2 in MVECs with different treatments. Control, untreated MVECs; exE2F2, E2F2-overexpressing MVECs; 221mimic, miR-221 mimic-treated MVECs; TCexo, exosomes derived from TCs; LPS, LPS-stimulated TCs; enJAK, TCs with JAK/STAT pathway enhancement; LPS/si221, LPS and miR-221 inhibitor-treated TCs. **c** Angiogenesis, proliferation, and migration assays in normal and E2F2-overexpressing MVECs incubated with different TCs-derived exosomes. Left: Normal MVECs; Right: E2F2-overexpressing MVECs. Control, MVECs incubated with exosomes derived from untreated TCs; LPS, MVECs incubated with exosomes derived from LPS-stimulated TCs; LPS-si221, MVECs incubated with exosomes derived from LPS and miR-221 inhibitor-treated TCs; 221mimic, MVECs incubated with exosomes derived from miR-221 mimic-treated TCs. exE2F2-Control, E2F2-overexpressing MVECs incubated with exosomes derived from untreated TCs; exE2F2-LPS, E2F2-overexpressing MVECs incubated with exosomes derived from LPS-stimulated TCs; exE2F2-LPS-si221, E2F2-overexpressing MVECs incubated with exosomes derived from LPS and miR-221 inhibitor-treated TCs; exE2F2-221 mimic, E2F2-overexpressing MVECs incubated with exosomes derived from miR-221 mimic-treated TCs. scale bar: 200 μm. *P < 0.05, **P < 0.01, ***P < 0.001

To validate E2F2 as a direct target of miR-221 and evaluate its role in mediating the angiogenic effects of TC-derived exosomes, a series of molecular and functional analyses were performed. Significant inhibition of E2F2 expression by miR-221 through binding to the wild-type (WT) E2F2 3’ UTR was demonstrated by dual-luciferase reporter assays, while mutation of the binding site abolished the inhibition (*P* < 0.01, Fig. 6a). Overexpression of E2F2 in MVECs significantly increased E2F2 levels, whereas the introduction of miR-221 mimic markedly reduced E2F2 expression in MVECs (all *P*-values were significant, Fig. 6b). Moreover, incubation of E2F2-overexpressing MVECs with exosomes derived from TCs following LPS stimulation or JAK/STAT signaling pathway enhancement significantly inhibited E2F2 expression, which was counteracted by the application of exosomes from TCs treated with LPS and miR-221 inhibitor (all *P*-values were significant, Fig. 6b). As shown in Fig. 6c, incubation with LPS-stimulated TCs-derived exosomes significantly promoted tube formation, proliferation, and migration in normal MVECs (all *P*-values were significant). Nonetheless, these effects were markedly reduced in MVECs incubated with exosomes from TCs under LPS stimulation and miR-221 inhibition (all *P*-values were significant, Fig. 6c). In E2F2-overexpressing MVECs, the E2F2-Control group exhibited significantly decreased angiogenic, proliferative, and migratory abilities compared to normal MVECs, while LPS stimulation of TCs in the E2F2-LPS group significantly alleviated this inhibition (all *P*-values were significant, Fig. 6c). Treatment with TCs-derived exosomes following LPS stimulation combined with miR-221 inhibition significantly reduced tube formation, proliferation, and migration (all *P*-values were significant, Fig. 6c). Furthermore, the E2F2-221mimic group in which cells incubated with miR-221 mimic-treated TCs-derived exosomes, exhibited a notable increase in these cellular activities in comparison with the E2F2-LPS-si221 group (all *P*-values were significant, Fig. 6c).

### TCs-derived exosomal miR-221 reduces lung inflammation in ARDS mice

**Fig. 7:**
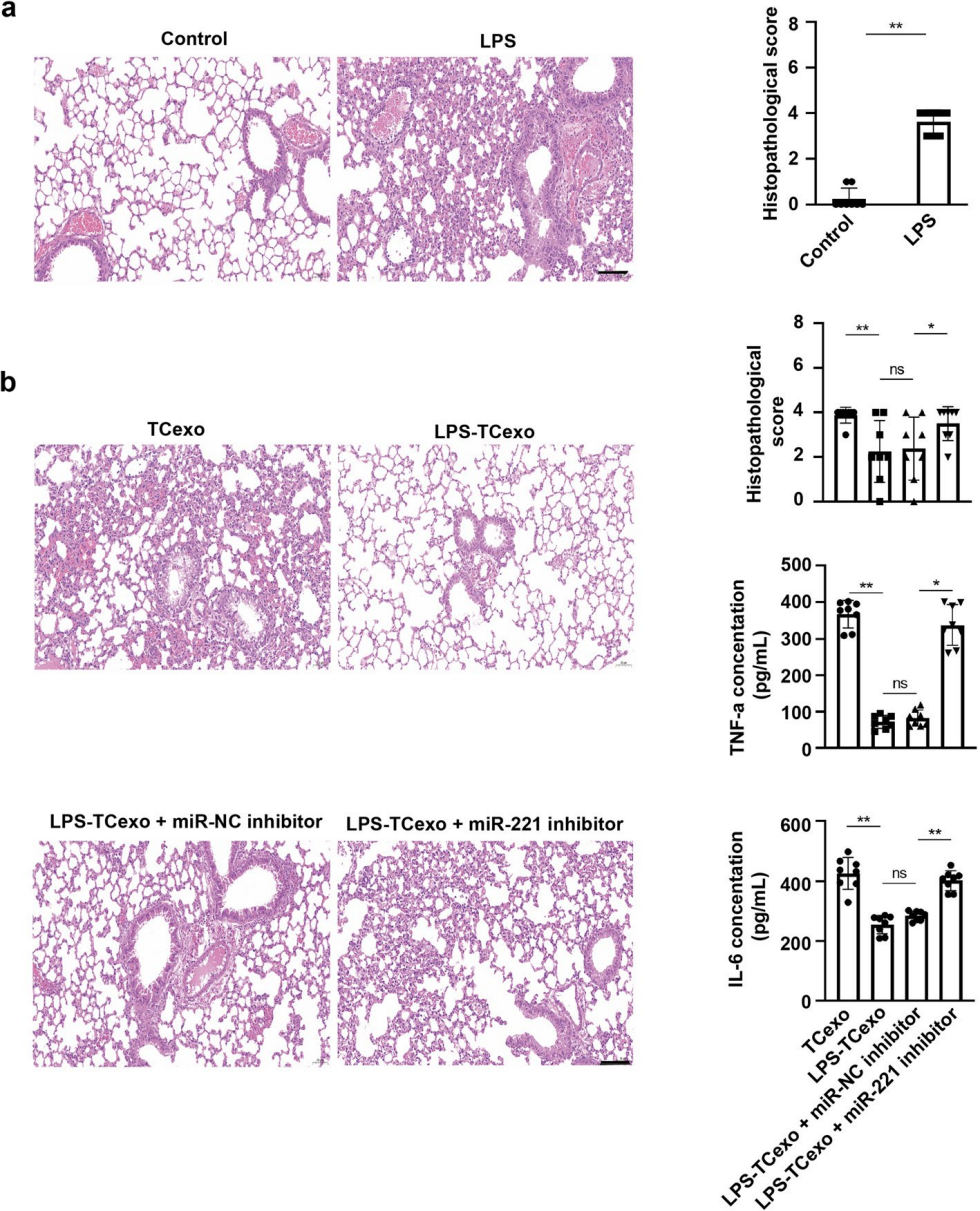
TCs-derived exosomal miR-221 reduces lung inflammation in ARDS mice. **a** Histopathological analysis of lung tissues from control and ARDS mice. Control, untreated mice; LPS, LPS-treated mice. scale bar: 50 μm. **b** Histopathological analysis of lung tissues and serum cytokine levels from ARDS mice treated with different TCs-derived exosomes. TC-exo, exosomes derived from TCs; LPS-TCexo, exosomes derived from LPS-stimulated TCs; LPS-TCexo + miR-NC inhibitor, exosomes derived from LPS and negative control-treated TCs; LPS-TCexo + miR-221 inhibitor, exosomes derived from LPS and miR-221 inhibitor-treated TCs. scale bar: 50 μm. n = 8. *P < 0.05, **P < 0.01

To evaluate the therapeutic potential of TC-derived exosomal miR-221 in vivo, we assessed the effects of various treatments on lung inflammation and tissue damage using established ARDS mice model. Histopathological analysis revealed significant lung tissue inflammation and damage in the LPS-treated group relative to the control group, confirming the successful establishment of ARDS mouse model (*P* < 0.01, Fig. 7a). Subsequently, ARDS mice treated with exosomes derived from LPS-stimulated TCs exhibited significantly lower histopathological scores and less severe inflammation compared to those treated with TCs-derived exosomes, which showed increased histopathological scores and more pronounced inflammation (*P* < 0.01, Fig. 7b). In contrast, the application of miR-221 inhibitor resulted in a marked elevation in histopathological scores (*P* < 0.05, Fig. 7b). Inflammatory cytokine levels in the plasma further supported these findings. Tumor necrosis factor-alpha (TNF-α) and interleukin-6 (IL-6) concentrations were significantly reduced with LPS-stimulated TCs-derived exosome treatment, while treatment with TCs-derived exosomes without LPS stimulation resulted in higher levels of these cytokines (all *P*-values were significant, Fig. 7b). A significant increase in TNF-α and IL-6 concentrations was observed with the additional application of miR-221 inhibitor, indicating heightened inflammation (all *P*-values were significant, Fig. 7b). Moreover, LPS-TCexo treatment significantly reduced pulmonary edema as evidenced by decreased lung wet-to-dry weight ratio and Evans blue extravasation (Fig. S3, S4). Importantly, survival analysis over 12 days demonstrated that LPS-TCexo treatment significantly improved survival rates in ARDS mice compared to both LPS and si221-LPS-TCexo groups (Fig. S5).

**Fig. 8:**
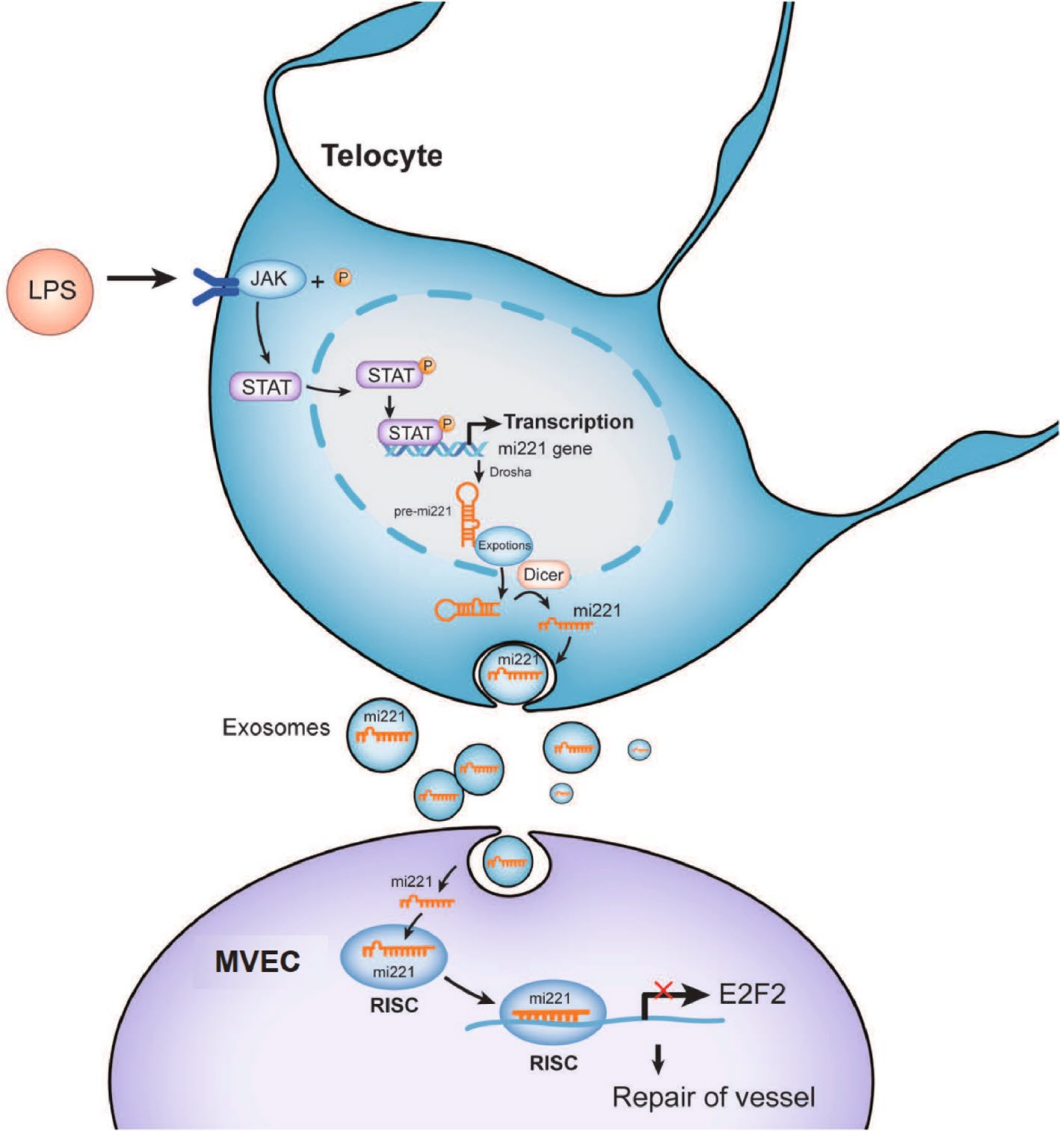
Schematic model of TCs-derived exosomes promoting MVEC angiogenesis through JAK/STAT-exosomal miR-221-5p-E2F2 axis

In summary, our results have demonstrated that LPS stimulation activated the JAK/STAT pathway in TCs, leading to the expression of miR-221 and the subsequent release of miR-221-enriched exosomes. These exosomes were taken up by vascular endothelial cells, where miR-221 inhibited E2F2 expression, thereby promoting angiogenesis (Fig. 8).

## Discussion

This study provides new insights into the reparative roles and mechanisms of telocyte-derived exosomal miRNAs in ARDS, reporting that TCs-derived exosomal miR-221 can significantly enhance angiogenesis and reduce inflammation in ARDS. Exosomes from LPS-stimulated TCs, enriched with miR-221, promoted MVECs functions, in which miR-221 induced by the JAK/STAT pathway activation inhibited E2F2 expression, thus facilitating MVECs angiogenesis, proliferation and migration. In ARDS mice, treatment with these exosomes decreased lung inflammation and damage, as shown by lower histopathological scores and diminished TNF-α and IL-6, effects reversed by miR-221 inhibition.

Endotoxin-induced ARDS has been reported to cause severe damage to both respiratory epithelial cells and underlying vascular endothelial cells [3]. TCs, distinct from mesenchymal stem cells and fibroblasts, play essential roles in cell–cell communication, angiogenesis and tissue repair [33]. In the present study, exosomes from LPS-stimulated TCs effectively reversed the angiogenic signaling impaired by LPS-induced inflammation, supporting the observation that TCs alleviate LPS-induced lung injury in mice through angiogenic factor release [18]. Notably, the significant upregulation of miR-221 in TCs-derived exosomes under LPS stimulation underscored the importance of miR-221 in vascular repair processes.

Non-coding miRNAs play roles in various pathological processes, including angiogenesis [34, 35]. MiR-21a-3p [36–38], miR-146a-5p [39], and miR-221-5p [40], were reported to be associated with the angiogenesis process. MiR-21a-3p and miR-221-5p were demonstrated to be involved in angiogenesis promotion in TCs [36–38]. We focused on miR-221-5p here due to its notable upregulation in TCs and derived exosomes as well as roles in promoting vascular endothelial cell angiogenesis. Knockdown of miR-221-5p in TCs led to a significant reduction in tube formation, proliferation, and migration of MVECs, supporting its protective effects on vascular repair, consistent with previous reports that miR-221-5p played critical roles in cancer, cardiovascular, and inflammatory diseases [40–42].

The JAK/STAT signaling pathway was identified as a crucial mediator in the production of miR-221 in TCs. Our data showed that LPS stimulation significantly activated the JAK/STAT pathway, leading to increased miR-221 expression in TCs and their exosomes. Inhibition of the JAK/STAT pathway effectively reduced miR-221 levels and attenuated the pro-angiogenic effects of exosomal miR-221, thereby highlighting the pathway’s role in regulating miR-221-mediated angiogenesis, which aligns with previous studies that have identified the JAK/STAT pathway as a key regulator of miRNA expression in inflammatory conditions [43].

E2F2, a transcription factor involved in cell cycle progression, apoptosis, and angiogenesis, was identified as a direct target of miR-221. The inhibition of E2F2 by miR-221 in MVECs promoted angiogenesis, as evidenced by increased tube formation, cell proliferation, and migration. These findings were consistent with previous studies highlighting E2F2’s essential roles in vascular integrity and angiogenesis [44, 45] thereby elucidating its regulatory mechanisms. Dual-luciferase reporter assays further confirmed the binding interaction between miR-221 and the E2F2 3’ UTR, revealing the molecular mechanism underlying miR-221-mediated angiogenesis.

In vivo studies using the ARDS mouse model reinforced the protective effects of TCs-derived exosomal miR-221. Mice treated with exosomes from LPS-stimulated TCs exhibited significantly reduced lung inflammation and tissue damage, as indicated by lower histopathological scores and decreased levels of inflammatory cytokines TNF-α and IL-6. These therapeutic effects were reversed upon miR-221 inhibition, underscoring the key roles of miR-221 in modulating inflammatory responses and promoting tissue repair.

Our findings highlight TCs-derived exosome therapy as a promising approach for ARDS, offering efficient lung distribution and biological stability without direct cell risks like immune response or tumorigenicity. The identified JAK/STAT-miR-221-E2F2 axis offers a specific target for therapy optimization. This method enables simultaneous modulation of angiogenic and inflammatory responses, essential for ARDS’s complex pathology, and may adapt to disease severity through TCs’ exosome release in response to inflammatory signals like LPS.

Two main aspects of the current study require further investigation. While we demonstrated the therapeutic potential of exosomal miR-221 and its role in promoting angiogenesis as one important mechanism for ARDS improvement, a more comprehensive analysis of the TCs-derived exosomal miRNA content is needed to fully understand additional protective mechanisms that likely exist. The LPS-induced ARDS model used in this study, while widely adopted, has inherent limitations in replicating the complex pathophysiology of clinical ARDS. The acute inflammatory response triggered by direct LPS administration may not fully reflect the diverse etiologies seen in clinical settings, and the high endotoxin concentrations used experimentally differ from physiological conditions. Future studies employing multiple models of lung injury would help validate these findings and strengthen their clinical relevance. Furthermore, exploring the roles of TCs-derived exosomes across ARDS of different etiologies and elucidating underlying mechanisms, including other potential pathways through which TC-derived exosomes may contribute to ARDS resolution, would provide valuable insights for further exploration of TCs’ therapeutic utility.

Overall, this study provided compelling evidence that TCs-derived exosomal miR-221, regulated by the JAK/STAT pathway, played important roles in promoting angiogenesis and reducing inflammation in ARDS through targeting E2F2, suggesting that targeting TCs and their exosomal miRNAs might be a promising therapeutic strategy for ARDS and potentially other inflammatory and vascular diseases. Moreover, the ability of TCs to secrete various reparative factors in response to inflammatory stimuli, such as LPS, underscores the potential in enhancing tissue repair in lung injury, laying a foundation for future clinical applications in pulmonary damage.

## Material and methods

### Experimental design

This study investigated the therapeutic potential of TCs-derived exosomal miR-221 in alleviating ARDS through promoting angiogenesis, proliferation, and migration in MVECs and reducing lung inflammation in ARDS mice. In vitro, experiments involved MVECs treatment with exosomes derived from LPS-stimulated TCs, with and without miR-221 inhibition, to assess proliferation, migration, and tube formation. In vivo, an ARDS mouse model was established through intratracheal LPS instillation, followed by exosome treatments, with subsequent histopathological analysis of lung tissues and measurement of plasma cytokine levels (TNF-α and IL-6). The role of the JAK/STAT pathway in the regulation of miR-221 and the inhibitory effect of miR-221 on E2F2 were investigated using qRT-PCR, western blotting, dual-luciferase reporter assays, and RNA in situ hybridization.

### Animal models

Eight-week-old male C57BL/6 mice, weighing 22 to 25 g, were purchased from Shanghai Jiesijie Company (Shanghai, China). The mice were randomly assigned to groups: Control, ARDS, Control with TCs-exosome (exo) treatment, ARDS with TCs-exo treatment, ARDS with negative control (NC) inhibited TCs-exo treatment, and ARDS with miR-221-5p inhibited TCs treatment. Under anesthesia (60 mg/kg sodium pentobarbital, Sinopharm Chemical Reagent Co., Shanghai, China), the mice received intratracheal instillations of either phosphate-buffered saline or LPS (5 mg/kg, Sigma, Germany) using 20-gauge catheters. Mice in the ARDS treatment groups were also injected via the tail vein with 100 μL of exosomes derived from TCs treated with either the NC or miR-221-5p inhibitor in the presence of LPS. 24 h post-treatment, the animals were sacrificed, and their lungs were harvested.

The study protocol was approved by the Animal Ethics Committee of Zhongshan Hospital, Fudan University (2023–214).

### Measurement of lung wet-to-dry weight ratio

The measurement of lung wet-to-dry weight ratio began with obtaining representative lung tissue samples from mice, followed by immediate wet weight measurement using an analytical balance. The samples were then placed in a drying oven at 60 °C for approximately 48 h until a constant weight was achieved. After the drying process, the dry weight was measured using the same analytical balance, and the wet-to-dry weight ratio was calculated by dividing the wet weight by the dry weight.

### Assessment of endothelial barrier integrity

To evaluate endothelial barrier integrity, 0.5% Evans Blue dye solution was administered to treated mice via tail vein injection at 2–3 ml/kg body weight, with blue coloration appearing in the skin within seconds to 1 min. After 0.5–1 h, the mice were euthanized and the target lung tissue was harvested and placed in a 1.5 ml centrifuge tube containing 1 ml PBS for rapid homogenization. The homogenate was centrifuged at 1000 g for 15 min at 4 °C, after which the supernatant was collected and mixed with an equal volume of trichloroacetic acid for 18–24 h incubation at 4 °C. Following centrifugation at 1000 g for 30 min at 4 °C, 1–2 ml of the resulting solution was analyzed spectrophotometrically at 620 nm. The Evans Blue content in lung tissue was then quantified using a standard curve generated from known concentrations of Evans Blue measured under identical conditions.

### Survival analysis

Using initial body weight at the start of the experiment as baseline, mouse body weight was recorded every 24 h. Mice exhibiting more than 30% weight loss from baseline were considered to have reached the experimental endpoint and were euthanized in accordance with animal ethics guidelines. The experiment lasted for 14 days, and survival data were analyzed using Kaplan–Meier survival curves.

### TCs and MVECs culture

Mouse primary pulmonary TCs were generously provided by Dr. Dongli Song. TCs were cultured in Dulbecco’s modified Eagle’s medium/F12 (DMEM/F12, Hyclone, Boston, MA) supplemented with 5% fetal bovine serum (FBS, Cellsera, Australia). Experiments involving LPS (0.1 μg/mL) were conducted in DMEM/F12 without FBS. The culture medium was collected from the culture dishes after 48 h of LPS stimulation.

For mouse vascular endothelial cells (MVECs), C57BL/6 mice were anesthetized and perfused with PBS via the heart to remove blood from the vasculature. Lungs were collected and washed in cold PBS, cut into 1–2 mm pieces, and incubated with collagenase I (1–2 mg/mL) in DMEM at 37 °C for 30–60 min with occasional agitation. Following collagenase treatment, the tissues were further digested with 0.25% trypsin–EDTA for 10 min, and the digestion was stopped with DMEM containing 10% FBS. The cell suspension was filtered through a 70 μm cell strainer to remove undigested tissue, centrifuged at 1200 rpm for 5 min, and the supernatant was discarded. Cells were resuspended in DMEM or EGM containing 10% FBS and 1% penicillin–streptomycin, and plated on collagen-coated culture dishes. Cultures were maintained at 37 °C in a 5% CO_2_ incubator. After 24–48 h, non-adherent cells were removed by changing the medium. Inhibition of JAK/STAT signaling pathway was performed with Ruxolitinib (HY-50856, MedChemExpress, USA) in accordance with manufacturer’s instructions. CDS nucleotide sequence (1332 nt) (shown in Supplementary Materials) of E2F2 was cloned into expression vector pLV-5HL.

### Real time cellular analysis

Real time cellular analysis (RTCA) using the Agilent xCELLigence system began by placing an E-Plate on the RTCA device cradle and adding 100 μL of sterile medium to each well, followed by a 30-min incubation for even distribution. Calibration was performed to ensure normal baseline impedance in all wells. MVEC cells were prepared by digesting, centrifuging, and resuspending to a concentration of 3 × 10^4^ cells/well, with viability confirmed above 95%. MVECs were then seeded into the calibrated E-Plate with 100 μL of the cell suspension per well and left to attach for 30 min. Initial readings were taken every 15 min for the first 2 h to monitor cell attachment. Following stable initial readings, treatments were performed. Measurements were set to taken every 15 min over a period of 24 h. Then, data were exported and analyzed to evaluate cell proliferation visualized as real-time curves.

### Exosome collection, isolation, purification and internalization experiments

TCs were cultured for 5 days. Subsequently, the existing medium was discarded, and the dish was rinsed 3 times with PBS to remove the residual exosomes. To collect exosomes, 10% exosome-free FBS DMEM was used. The exosomes present in the medium were then isolated by ultracentrifugation [47]. Specifically, the process involved initial centrifugation steps of 300 × g for 5 min, 2,000 × g for 5 min, and 12,000 × g for 30 min to remove cell debris and large vesicles from the supernatant, which was then filtered through a 0.22-μm sieve. Following this, the exosomes were isolated by ultracentrifugation at 110,000 × g, washed with PBS, and collected at 110,000 × g. The resulting exosomes were resuspended in 200 μL of PBS. Additionally, exosomes were also isolated using a precipitation method with ExoJuice (ExonanoRNA, Foshan, China) according to the manufacturer’s instructions. Briefly, the cell culture medium was centrifuged at 12,000 × g for 30 min, after which the supernatant was transferred to an ultracentrifuge tube, and 1 mL of ExoJuice was added to the bottom of the tube. The mixture was then centrifuged at 100,000 × g for 70 min. The first 500 μL of liquid from the bottom of the tube was carefully discarded, and the subsequent 300 μL of liquid, containing the purified exosomes, was carefully collected and retained.

The successful isolation was confirmed by transmission electron microscopy showing typical exosomal morphology (Fig. S1).

Following a 24-h incubation of PKH26-labeled exosomes with MVECs, the cells were fixed with 4% paraformaldehyde. Subsequently, 4′,6-diamidino-2-phenylindole (DAPI) was utilized to stain the nuclei, and an Olympus microscope (Japan) was employed to detect the PKH267-labeled exosomes.

### Gene expression profiling analysis

Gene expression profiling of both miRNA and mRNA was conducted using the Agilent Microarray Scanner (Cat # G2565CA, Agilent Technologies, Santa Clara, CA). Data normalization was performed with the *AgiMicroRna* package [48]. Differentially expressed genes (DEGs) were identified based on an adjusted *P*-value of less than 0.05 and further analyzed using the *limma* package [49]. Heatmaps were created with the *ggplot2* package. The *algorithm* of *Miranda* v3.3a [50] and *TargetScan* v8.0 [51] were utilized to identify potential miRNA target genes. Genes identified by both software were selected for further analysis. GO ontology functional analysis of the genes was conducted using the *STRING* database [52]. Phenotype Screening were further conducted using two proliferation-associated datasets of HALLMARK_ANGIOGENESIS and HALLMARK_G2M_CHECKPOINT form MSigDB database [53].

### RNA in situ hybridization and fluorescence staining

RNA in situ hybridization (RNA ISH) was used to detect miR-221 molecule in MVECs or TCs. First, MVECs or TCs were fixed with 4% paraformaldehyde for 10–20 min and washed with PBS. Then, cells were treated with proteinase K (ST533, Beyotime Biotechnology, China), followed by PBS washes. Afterward, cells were dehydrated through an ethanol gradient and air-dried. Pre-hybridization buffer was applied, followed by hybridization buffer containing miR-221 probes, and incubated at 37 °C overnight. Post-hybridization, samples were washed with hybridization wash buffer at 37 °C. For fluorescence detection, cells were blocked with 1% BSA in PBST (PBS + 0.1% Tween 20) for 30 min at room temperature. The fluorophore-conjugated detection probe specific for miR-221 was diluted in hybridization buffer according to manufacturer’s instructions and samples were incubated with the detection probe for 1 h at room temperature in a humidified chamber protected from light. After washing three times with PBST, nuclei were counterstained with DAPI (1:5000 dilution) for 5 min. Slides were mounted using anti-fade mounting medium. Fluorescence microscopy was then performed for detection, and images were captured under a microscope to assess the localization and intensity of hybridization signals. Fluorescence intensity was quantified using ImageJ software from at least five random fields per sample.

### Dual luciferase assay

The pGL3 reporter vector (Promega, Madison, WI) was used to generate the plasmids pGL3-WT-E2F2-3′-UTR and pGL3-Mut-E2F2-3′-UTR. Human embryonic kidney cells were co-transfected with pGL3-E2F2-3′-UTR (WT or Mut) and the miR-221-5p mimic or NC with Lipofectamine 2000 reagent (Thermo Fisher Scientific, Carlsbad, CA). After a 24-h incubation, luciferase activity was measured using the Dual-Luciferase Reporter Assay System (Promega, Madison, WI) according to the manufacturer’s protocol.

### Tissue preparation and HE staining

Lung tissues were fixed in 10% formalin solution and embedded in paraffin. Each tissue was sectioned at a thickness of 5 μm and stained with hematoxylin–eosin (HE, Beyotime, Shanghai, China) following the manufacturer’s instructions.

Histopathological scoring was performed by two independent blinded pathologists using a 0–4 scale (0 = normal, 1 = mild, 2 = moderate, 3 = severe, 4 = very severe) evaluating five parameters: (1) alveolar wall thickening (multilayered cells on alveolar walls), (2) hemorrhage (extravasation of red blood cells), (3) congestion (intravascular accumulation of red blood cells), (4) necrosis (nuclear fragmentation, pyknosis, and lysis), and (5) inflammatory cell infiltration. The total score represents the sum of all parameters (maximum 20).

### Statistical analysis

Data were presented as the means ± SDs and analysis were conducted by one-way analysis of variance (ANOVA) and Tukey’s multiple comparisons test. *P*-value of < 0.05 was considered statistically significant. All statistical analyses were performed with *GraphPad Prism* v7.04 (GraphPad, San Diego, CA).

## Supplementary Information

Supplementary Material 1.

## Data Availability

The original research data presented in this study are included in the article and Supplementary Material. The miRNA sequencing data have been deposited in the NCBI Sequence Read Archive (SRA) (accession number PRJNA1186347) (https://www.ncbi.nlm.nih.gov/sra/PRJNA1186347). Further inquiries can be directed to the corresponding author.
