# Supplementary material for: Telocyte-derived exosomes promote angiogenesis and alleviate acute respiratory distress syndrome via JAK/STAT-miR-221-E2F2 axis
Source: Mol Biomed. 2025 Apr 8;6:21. doi: 10.1186/s43556-025-00259-6 (PMC11979044; doi:10.1186/s43556-025-00259-6)
Supplement: Supplementary file 1 — Supplementary Material 1. [file 43556_2025_259_MOESM1_ESM.docx]

**Telocyte-derived exosomes promote angiogenesis and alleviate acute respiratory distress syndrome via JAK/STAT-miR-221-E2F2 axis**

Rongrong Gao^1,2†^, Xu Zhang^3,4†^, Huihui Ju^1†^, Yile Zhou^1^, Luoyue Yin^1^, Liuke Yang^5^, Pinwen Wu^6^, Xia Sun^7*^ and Hao Fang^1,7*^

^1^ Department of Anesthesiology, Zhongshan Hospital, Fudan University, Shanghai, China.

^2^ Clinical Center for Biotherapy at Zhongshan Hospital, Fudan University, Shanghai, China.

^3^ NHC Key Lab of Reproduction Regulation, Shanghai Engineering Research Center of Reproductive Health Drug and Devices, Shanghai Institute for Biomedical and Pharmaceutical Technologies, Shanghai, China.

^4^ Shanghai-MOST Key Laboratory of Health and Disease Genomics, NHC Key Lab of Reproduction Regulation, Shanghai Institute for Biomedical and Pharmaceutical Technologies, Shanghai, China.

^5^ College of Plant Protection, Nanjing Agricultural University, Nanjing 210000, China.

^6^ Department of Anesthesiology, Minhang Hospital, Fudan University, Shanghai, China.

^7^ Department of Anesthesiology, Shanghai Geriatric Medical Center, Shanghai,China.

Author e-mails: Rongrong Gao ([lsq274241075@163.com](mailto:lsq274241075@163.com)), Xu Zhang ([ZhangXu5920@outlook.com](mailto:ZhangXu5920@outlook.com)), Huihui Ju ([juhui927@126.com](mailto:juhui927@126.com)), Yile Zhou ([yile727@hotmail.com](mailto:yile727@hotmail.com)), Luoyue Yin ([2695409795@qq.com](mailto:2695409795@qq.com)), Liuke Yang（[LKYeNJ23@126.com](mailto:LKYeNJ23@126.com)) , Pinwen Wu (pw_1976@163.com)

†These authors contributed equally to this work

Correspondence: Hao Fang [drfanghao@163.com](mailto:drfanghao@163.com)

Co-correspondence: Xia Sun [18017312998@163.com](mailto:18017312998@163.com)

**Supplementary Materials**

**Material and methods**

**Cell migration, wound-healing, and tube formation assays**

In the cell migration assay, the upper chamber was initially incubated with serum-free DMEM for 2 h to rehydrate the chamber. DMEM supplemented with 10% FBS was then added to the lower chamber. MVECs were either treated with exosomes (10 µg of exosomes resuspended in 100 µL of PBS and added with 1 × 10^5^ MVECs) or transfected with miRNA mimics or plasmids for 24 h. Subsequently, MVECs were collected, diluted with serum-free DMEM (2 × 10^5^ cells in 100 µL), and added to the upper chamber. Following a 24-h incubation, five random visual fields (× 200) were counted under a light microscope. Each experiment was performed in triplicate.

For the wound-healing assay, MVECs were treated with exosomes (10 µg of exosomes resuspended in 100 µL of PBS and added with 1 × 10^5^ MVECs) or transfected with miRNA mimics or plasmids for 24 h. Then, MVECs were harvested and seeded into a 6-well plate at a density of 5× 10^5^ cells per well. A sterile 200-µL pipette tip was then used twice to make a scratch, and floating cells were removed by rinsing with PBS. Images of the scratches were captured using an inverted microscope at 100 × magnification at 0 and 24 h post-scratch. The average distance of the healed wound area was measured by comparing the images taken at 24 h and 0 h using an Olympus IX71 microscope (Olympus).

In matrigel tube formation assay, MVECs were treated with exosomes (10 µg of exosomes resuspended in 100 µL of PBS and added with 1 × 10^5^ MVECs) or transfected with miRNA mimics or plasmids for 24 h. Following this treatment, MVECs were harvested, resuspended in serum-free DMEM, and seeded into 96-well plates (20,000 cells per well) that were pre-coated with growth factor-reduced basement membrane matrix (BD Biosciences). The plates were then incubated at 37°C for 6-8 h. Tube formation was visualized using an inverted microscope, and enclosed networks of tube structures from three randomly selected fields were recorded under a light microscope.

**Quantification of mRNA and miRNA**

Total RNA was extracted from cultured TCs or MVECs using TRIzol (Takara, Shiga, Japan) according to the manufacturer's instructions. MiRNAs were reverse transcribed using the Bulge-Loop miRNA qRT-PCR Starter Kit (Ribobio, Guangzhou, China), while mRNAs were reverse transcribed to complementary DNA (cDNA) using the PrimeScript RT Reagent Kit with gDNA Eraser (Takara, Shiga, Japan). The expression levels of miR-146a-5p, miR-155-5p, miR-21a-3p, miR-5100, miR-221-5p, miR-7a-5p, and mRNAs were quantified by quantitative real-time polymerase chain reaction (qPCR) on a Bio-Rad IQ5 real-time PCR system, using U6 and GAPDH as the housekeeping genes for miRNAs and mRNAs, respectively. MiRNA PCR was conducted with the Bulge-Loop miRNA qRT-PCR Starter Kit, Bulge-Loop mmu-miR-146a-5p Primer Set, Bulge-Loop mmu-miR-155-5p Primer Set, Bulge-Loop mmu-miR-21a-3p Primer Set, Bulge-Loop mmu-miR-5100 Primer Set, Bulge-Loop mmu-miR-221-5p Primer Set, Bulge-Loop mmu-miR-7a-5p Primer Set (Ribobio, Guangzhou, China). MRNA primers were synthesized by Sangon (Shanghai, China). The following primers were used in quantification of mRNA and miRNA: miR-146a-5p sense primer: 5’- AGCTCTGAGAACTGAA-3’, antisense primer: 5’-ACAGGTCTGACATTGA-3’, miR-155-5p sense primer: 5’-GTGATAGGGGTTTTGG-3’, antisense primer: 5’-GGAGTCAGTCAGAGGC-3’, miR-21a-3p sense primer: 5’-TACCACCTTGTCGGATAG-3’, antisense primer: 5’- CTGCTGTTGCCATGAGAT-3’, miR-5100 sense primer: 5’- GTGGGAGGGAGGACTTGGGAACTGA-3’, antisense primer: 5’- GTCGGGAAGAGGCACCGCTGGGATT-3’, miR-221-5p: 5’- CATGAACCTGGCATACA-3’, antisense primer: 5’-GAAACCCAGCAGACAAT-3’, miR-7a-5p sense primer: 5’- CAGGCCACTCTACAGGACA-3’, antisense primer: 5’-AGGAACATGAGGAAGGTGTGAA-3’, E2F2 sense primer: 5’-GAAGACACGCTATGACACG-3’, antisense primer: 5’- GGGTAACCGACAAATGC-3’, MEIS1 sense primer: 5’- TTGGCACAAGATACGGGACT-3’, antisense primer: 5’-TGACTGCTCGGTTGGACTG-3’, SUV39H1 sense primer: 5’-GTAGGCTTGTCTTAGATGGAGGTTGG-3’, antisense primer: 5’- CCTGCTGAGTGGTAGGAGTGGGATA-3’.

**Lung wet-to-dry weight ratio and Evans blue extravasation**

We conducted experiments examining lung wet-to-dry weight ratio and Evans blue extravasation to address the concerns regarding endothelial barrier integrity. Our results showed that LPS treatment significantly increased the wet-to-dry ratio compared to the control group (*P*<0.001), indicating severe pulmonary edema. Treatment with LPS-TCexo demonstrated the most significant improvement (*P*<0.001), while miR-221 inhibition partially reversed this protective effect (Fig. S2). These findings were further validated by Evans blue assays, where ARDS mice showed significantly increased dye content compared to controls (*P*<0.001). LPS-TCexo treatment markedly reduced dye extravasation (*P*<0.001).

**Survival experiments in evaluating ARDS interventions**

We have conducted survival experiments to strengthen our conclusions about the therapeutic potential of TC-derived exosomal miR-221 in ARDS treatment. For the survival study, we employed the LPS-induced ARDS mouse model as described in our original methods section. The results demonstrated that mice in the LPS-TCexo group exhibited significantly improved survival compared to both the LPS group and si221-LPS-TCexo group (*P*<0.001). Notably, the survival benefit was suppressed with miR-221 inhibition, while the control siRNA maintained therapeutic efficacy similar to the LPS-TCexo group (Fig. S4).

**Real-time cell analysis**

Regarding the demonstration of comparable initial cell density and growth status across experimental groups, we have performed real-time cell analysis (RTCA) to monitor MVEC growth dynamics under different treatment conditions over 72 hours. As shown in Figure S5, the data demonstrated consistent initial seeding density (0.5×10^5^ cells) across all experimental groups at 0 h and similar growth trajectories during the initial 24-hour period before formal testing. The comparable viability and growth patterns across control and treatment groups confirm that the differences observed in our functional assays reflect true biological responses to the various treatments rather than artifacts from differing initial conditions (Fig. 2D; Fig. 3D; Fig. 4D; Fig. 5E; Fig. 6C).

**Supplementary Figures**

**1. TCs-derived exosomes**


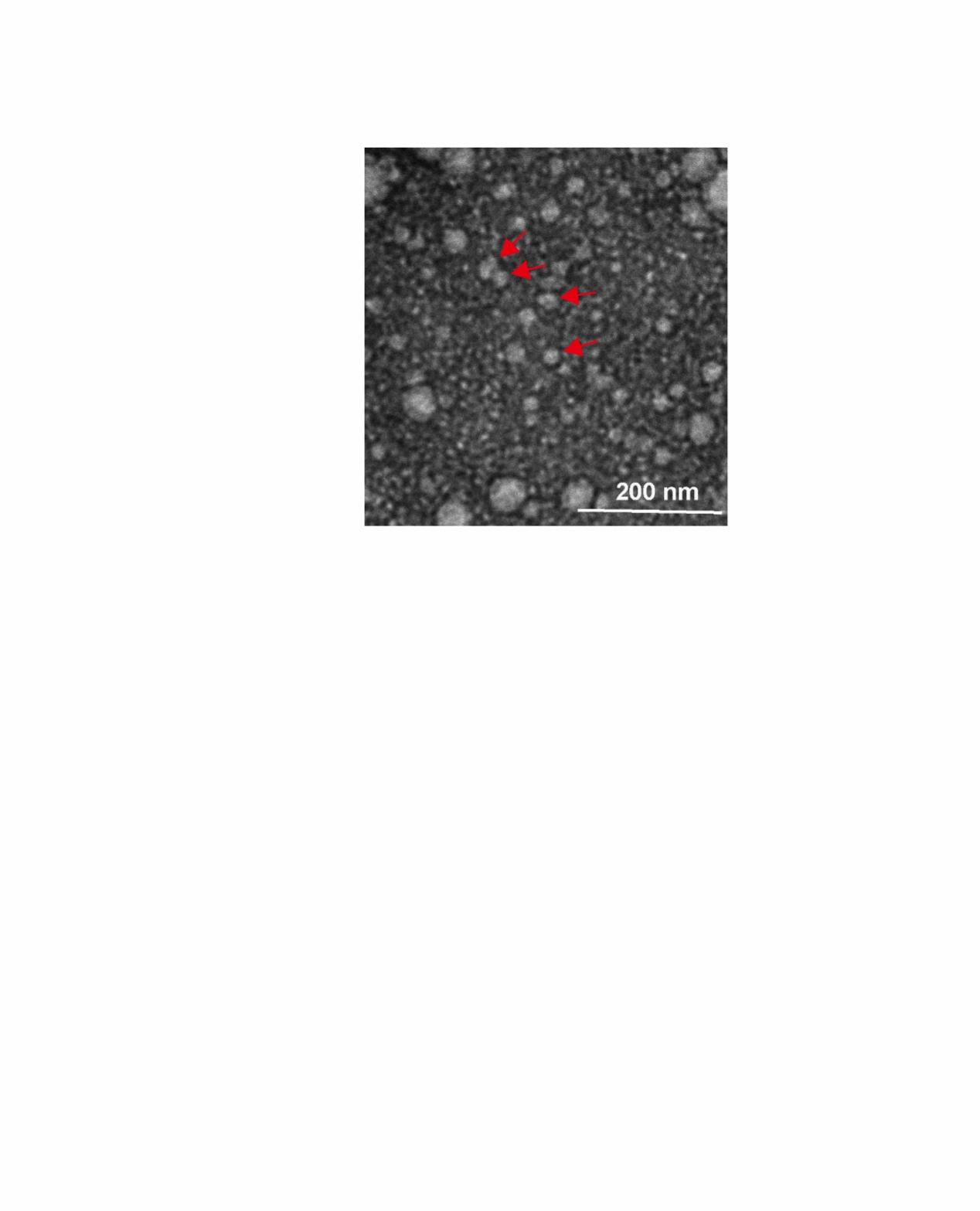


**Fig, S1** TEM images of TCs-derived exosomes. The red arrows indicate representative exosomes with well-preserved membrane integrity. scale bar: 200 nm.The transmission electron microscopy (TEM) images of TCs-derived exosomes were provided in Figure S1. The TEM images clearly show that the isolated vesicles display typical exosomal morphology, which, combined with the Western blot data showing exosomal markers (CD9, CD81, and CD63) in Figure 2a, confirms the successful isolation and characterization of TCs-derived exosomes.

**2. Endothelial barrier integrity:**


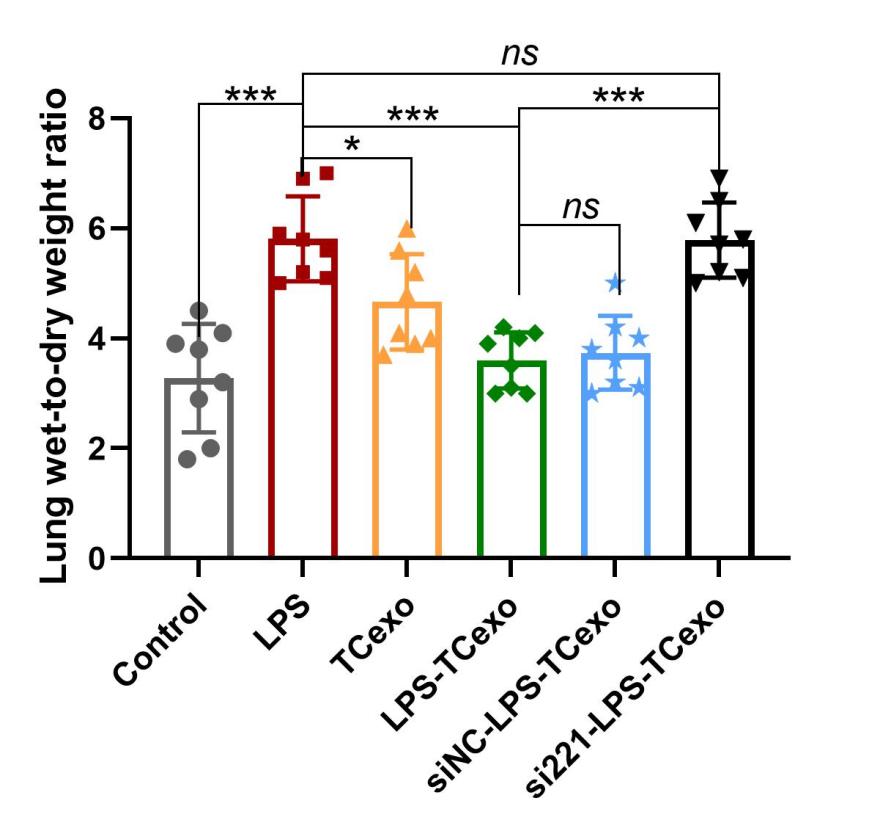


**Fig. S2** Lung wet-to-dry weight ratio analysis in ARDS mice treated with TCs-derived exosomes. Groups include Control (untreated), LPS (LPS-induced ARDS), TCexo (TCs-derived exosomes), LPS-TCexo (exosomes derived from LPS-stimulated TCs), siNC-LPS-TCexo (LPS-stimulated TCs-derived exosomes with negative control), and si221-LPS-TCexo (LPS-stimulated TCs-derived exosomes with miR-221 inhibition). Data are presented as mean±SD. **P*<0.05, ***P*<0.01, ****P*<0.001, ns: not significant. n=8 per group.

1. **miR-221 inhibition partially abolished this effect：**


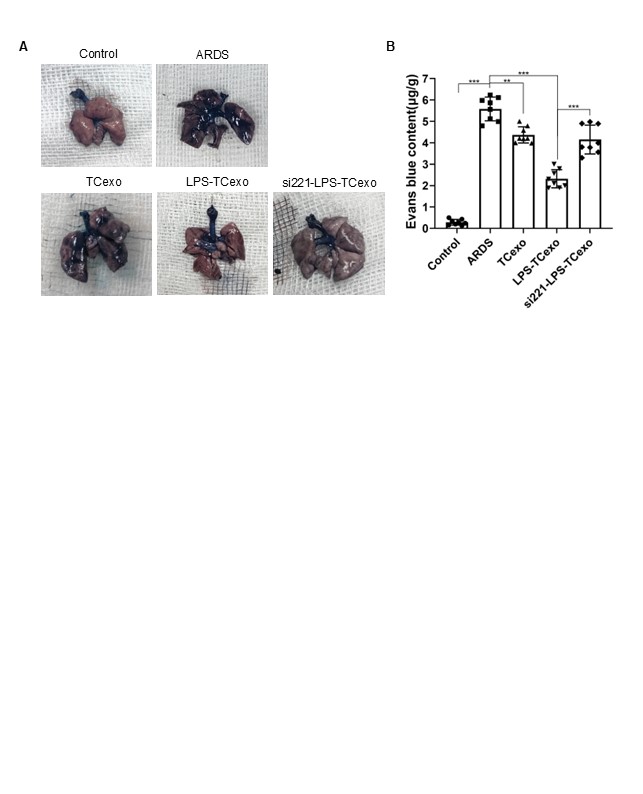


**Fig. S3** Analysis of pulmonary vascular permeability in ARDS mice. (A) Representative images of lung tissues after Evans blue dye administration in different treatment groups. Groups include Control (untreated), ARDS (LPS-induced), TCexo (TCs-derived exosomes), LPS-TCexo (LPS-stimulated TCs-derived exosomes), and si221-LPS-TCexo (LPS-stimulated TCs-derived exosomes with miR-221 inhibition). (B) Quantification of Evans blue content in lung tissue (μg/g) from different treatment groups. Data are presented as mean ± SD. ***P*<0.01, ****P*<0.001. n=8 per group.

1. **Survival analysis of different treatment groups in ARDS mice**


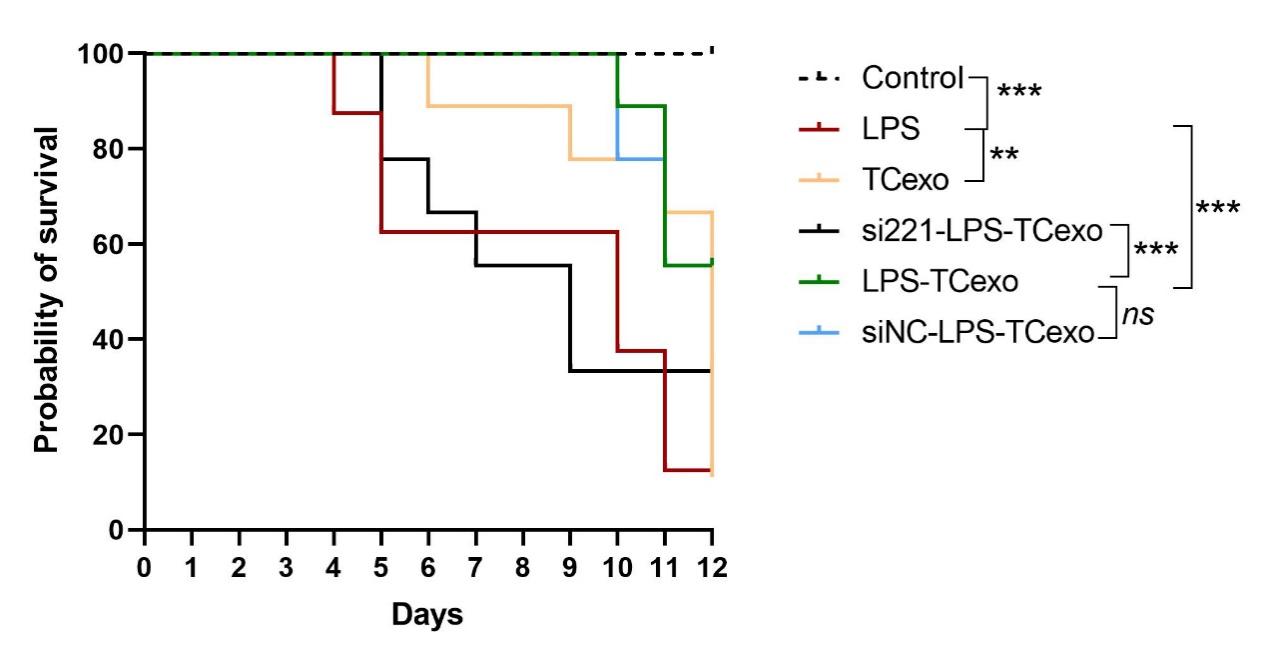


**Fig. S4** Survival analysis of different treatment groups in ARDS mice. The survival curves show the probability of survival over 12 days for different experimental groups. Groups include Control (untreated), LPS (LPS-induced ARDS), TCexo (TCs-derived exosomes), LPS-TCexo (exosomes derived from LPS-stimulated TCs), siNC-LPS-TCexo (LPS-stimulated TCs-derived exosomes with negative control), and si221-LPS-TCexo (LPS-stimulated TCs-derived exosomes with miR-221 inhibition). Data are presented as mean±SD. ***P*<0.01, ****P*<0.001, ns: not significant. n=8 per group.

**5.Growth curves showing cell density and proliferation of MVECs**


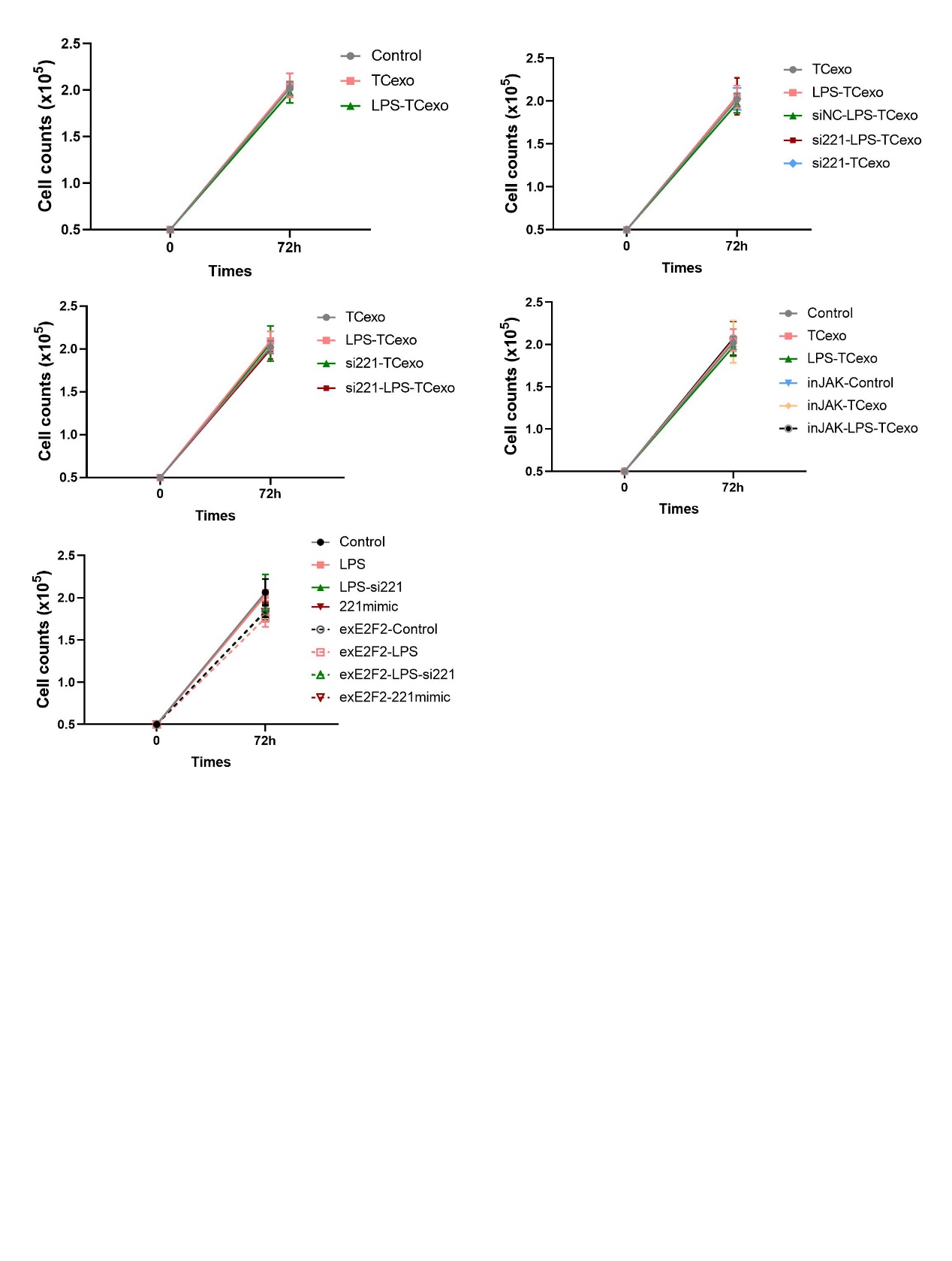


**Fig. S5** Growth curves showing cell density and proliferation of MVECs under different treatment conditions over 72 hours assessed by RTCA.

**Nucleotide sequence:**

CDS nucleotide sequence (1332 nt) of E2F2 was cloned into expression vector pLV-5HL:

ATGCTGCGCGCGCCGCGGACCCTGGCTCCGGCCACGGCGCAACCTACAAAGAGCTTGCCGGCGCTGAACCCCACCGAGCTGTGGCCTTCGGGTCTGAGCAGCCCCCAGCTCTGCCCGGCCACCACCGCCACCACCTACTACACTTCGCTTTACACGCAGACGGTGCCTTCCTCTGTGGCGCTGGGCACCTGCCTCGACGCCACTCCCCACGGACCCGAGGGCCAAATTGTGCGATGTGCACCCGCAGGCCGGCTGCCGGCCAAAAGGAAGTTGGACCTGGAGGGCATTGGGAGGCCTACGGTCCCTGAATTCCGGACCCCCAAGGGGAAGTGCATCCGCGTGGATGGTTTGCCAAGCCCCAAAACCCCCAAGTCTCCTGGGGAGAAGACACGCTATGACACGTCGCTGGGGCTCCTGACCAAGAAGTTCATTTACCTCCTGAGCGAGTCGGAGGATGGAGTCCTGGACCTGAACTGGGCAGCCGAGGTGCTGGATGTGCAAAAGCGGCGCATCTATGACATCACCAACGTGCTGGAGGGTATCCAGCTCATCCGCAAGAAGTCCAAAAACAACATCCAGTGGGTAGGCAGGGAACTATTTGAAGACCCCACCCGACCCTCCAGGCAGCAGCAGTTGGGGCAGGAGCTGAAGGAGCTGATGAATGCCGAGCAGACCTTGGACCAGCTCATTCAGAGTTGCTCCCTGAGCTTCAAGCACCTGACCGAAGATAATGCCAACAAGAAACTGGCCTATGTGACCTACCAGGATATCCGTGCCGTAGGCAACTTCAAGGAGCAGACAGTGATTGCGGTCAAGGCCCCACCACAGACAAGATTGGAAGTGCCGGACAGGGCCGAGGAGAACCTGCAGATTTATCTAAAGAGTACCCAAGGCCCCATTGAAGTCTACCTGTGCCCAGAGGAGGGGCAGGAGCCAGACAGTCCTGCCAAGGAGGCGCTCCCCTCCACCTCTGCCCTCAGCCCCATTCCTGACTGCGCTCAGCCGGGCTGCAGCACTGACTCTGGGATCGCAGAGACCATAGAGCCTTCAGTACTGATACCCCAGCCGATACCACCGCCTCCTCCACCACCACTGCCGCCAGCCCCATCCCTCGTCCCCTTGGAAGCCACTGACAACATGCTGGAGCTGTCACACCCTCTTCTACAACAGACTGAGGACCAGTTCCTGTCCCCAATCCTGGCGGCCAACTCCCCCCTGATCAGCTTCTCCCCGCCCTTGGACCAGGACGAATACCTGTGGGGCATGGACGAGGGGGAAGGCATCAGTGACCTCTTCGACTCCTATGACCTTGGGGACCTGTTGATTAATTGA
